# Supplementary material for: Efficient biosynthesis of nucleoside cytokinin angustmycin A containing an unusual sugar system
Source: Nat Commun. 2021 Nov 17;12:6633. doi: 10.1038/s41467-021-26928-y (PMC8599513; doi:10.1038/s41467-021-26928-y)
Supplement: Supplementary file 1 — Supplementary Information [file 41467_2021_26928_MOESM1_ESM.pdf]

Efficient biosynthesis of nucleoside cytokinin Angustmycin A containing an unusual sugar system

Yu *et al.*

Supplementary Method 1. Bioassay of angustmycins.
For angustmycin bioassay, *Mycobacterium smegmatis* mc^{2}155 was used as indicator strain. After incubation in Luria-Bertani medium at 37°C for 6 h, the bacterial cultures (75 mL, OD_{600}=0.8) was mixed into melted Luria-Bertani ager medium (30 mL). Related fermentation broth (50 μL) was added into the oxford cup, and the bioassay plate was cultivated at 37°C for 12 h to observe the antibacterial zone.

Supplementary Method 2. Extraction and purification of 1 and 2.
The fermentation method for M1154::pCHW501, M1154::pCHW501*ΔagmF*, and M1154::pCHW501*ΔagmR* was identical to that as described above. The cultured broth was acidified to pH 5.0 by oxalic acid before filtration. The M1154::pCHW501 and M1154::pCHW501*ΔagmF* filtrates were collected, and blended with an equal volume of methanol. After removal of precipitant, the collected supernatant (water-methanol mixture) was condensed and further dried by rotary evaporation. The dried residues were re-dissolved in methanol, and prepared by HPLC after passing through a 0.22 μm filter. NMR analysis of **1** and **2** were performed on Agilent DD2 400 MHz NMR spectrometer using DMSO as solvent.

Supplementary Method 3. Chemical structure identification of 1 and 2.
UV/Vis spectrum of **2** exhibited the characteristic absorption with λ_{max} at 257 nm; ESI-HRMS spectrum of **2** showed a quasi-molecular ion peak at m/z 298.1144 (calcd. for $C_{11}H_{15}N_5O_5^+$, m/z 298.1144 $[M+H]^+$). Detailed information of the 1D and 2D NMR was described in Supplementary Fig. 4 and 5, and Supplementary Table 1. Analysis of the 1D and 2D NMR data led to the identification of **2** structure.

UV/Vis spectrum of **1** exhibited the characteristic absorption with λ_{max} at 257 nm; ESI-HRMS spectrum of **1** showed a quasi-molecular ion peak at m/z 280.1040 (calcd. for $C_{11}H_{13}N_5O_4^+$, m/z 280.1040 $[M+H]^+$). Detailed information of the 1D and 2D NMR was described in Supplementary Fig. 6 and 7, and Supplementary Table 2. Analysis of the 1D and 2D NMR data led to the identification of **1** structure.

Supplementary Method 4. Overexpression and purification of the target proteins.
Expression and purification of AgmA, AgmB, AgmC, AgmD, AgmE, AgmF, and AlsE were carried out in a similar protocol. The engineered structural gene was cloned into pET28a and then transformed into *E. coli* BL21(DE3) (*agmC* was cloned into pSJ8). The overnight culture was inoculated into 500 mL of liquid culture, and the cells were grown at 37°C in Luria-Bertani medium with kanamycin (50 μg/mL) or ampicillin (100 μg/mL for pSJ8) until an OD_{600} of 0.6-0.8 and then induced with 0.1 mM isopropyl-β-D-1-thiogalactopyranoside. The culture was further incubated at 18°C for 18-20 h and centrifuged at 6,000 r/min for 5 min at 4°C, and the pellet was resuspended in 30 mL of lysis buffer (25 mM Tris-Cl, 150 mM NaCl, pH 7.0 for AgmB and AgmD, pH 7.5 for AgmA, AgmC, AgmE, AgmF, and AlsE). Cellular

debris was removed by centrifugation at 12,000 r/min for 40 min at 4°C. The supernatant was incubated with 1 mL of Ni-NTA agarose resin (QIAGEN) and was run through a gravity flow column. The protein was washed with washing buffer (25 mM Tris-Cl, 150 mM NaCl, and 20 mM imidazole) and eluted with elution solution (25 mM Tris-Cl, 150 mM NaCl, and 200 mM imidazole). Purified proteins were concentrated and buffer-exchanged into protein stock buffer (25 mM Tris-Cl, 150 mM NaCl, and 10% glycerol, pH 7.0 for AgmB and AgmD, pH 7.5 for AgmA, AgmC, AgmE, AgmF, and AlsE) using Amicon Ultra filters. The final proteins were flash-frozen in liquid nitrogen and stored at -80°C.

Supplementary Method 5. Heterologous expression of the related genes for 1 and 2 biosynthesis in *E. coli*.

The structural genes for **1** biosynthesis involving *agmD* (*alsE*), *agmC*, *agmA*, *agmE*, *agmB*, and *agmF* were cloned into pETDuet (*agmD*/*alsE* and *agmC*), pCDFDuet (*agmA* and *agmE*), and pRSFDuet (*agmB* and *agmF*), respectively, and introduced into *E. coli* GYJ23. The **2** targeted-accumulating strain harbored pETDuet (*agmD*/*alsE* and *agmC*), pCDFDuet (*agmA* and *agmE*), and pRSFDuet (*agmB*). As for the fermentation, the various co-expression strains were cultured at 37°C in Luria-Bertani for around 10 h to make consistency of the OD_{600} value for related strains. Followed by inoculating 5 mL fresh cells into 50 mL (50/500) fermentation medium (containing 0.3 mM IPTG, 50 mg/L antibiotics)[1]. During the fermentation process (180 r/min, 30°C), the cultures were sampled at 24 h, 48 h, 72 h, or 96 h, respectively. For HPLC analysis, the fermentation broth was processed by adding oxalic acid till pH 5.0. HPLC analysis was performed using Shimadzu LC-20AT equipped with C18 column (Diamonsil, 5 μm, 4.6 × 250 mm) under 0.15% aqueous formic acid (95%): methanol (5%) at a flow rate of 0.5 mL/min over 30 min. Detection wavelength was 254 nm.

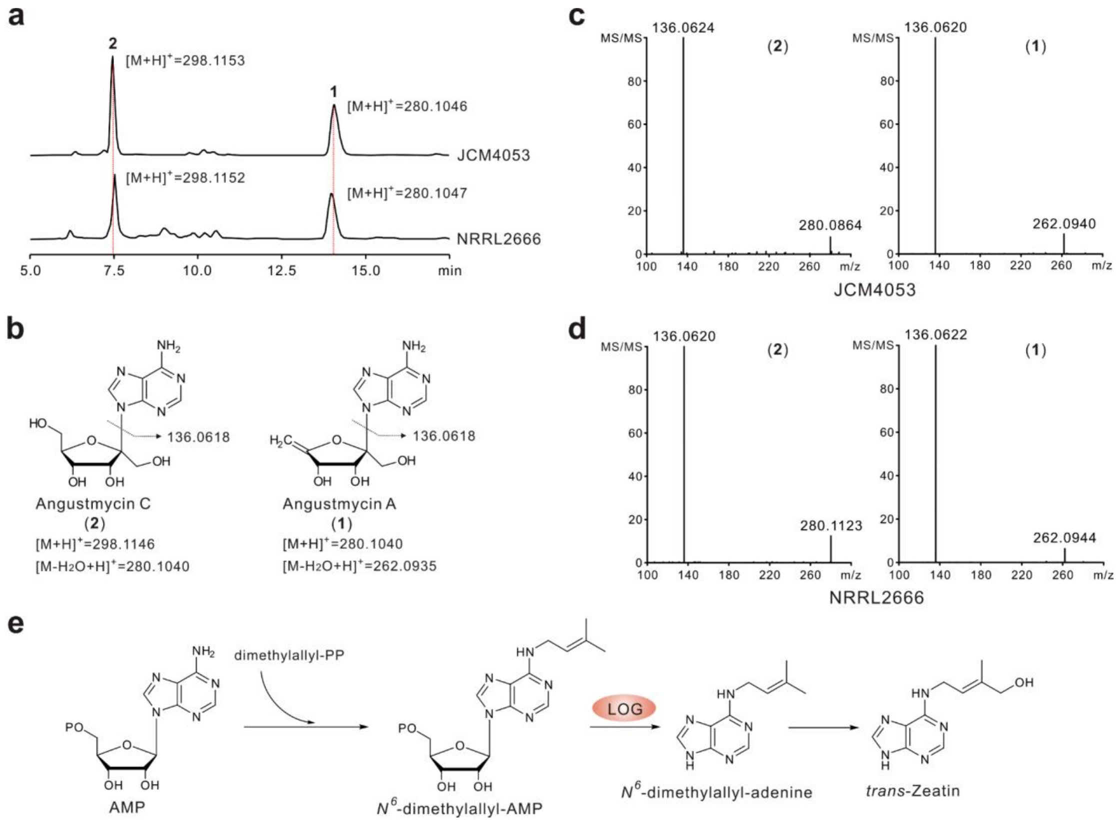

Supplementary Fig. 1. Metabolites analysis of the two angustmycin producing strains.

a, HPLC analysis (λ=254 nm) of the metabolites produced by related strains. JCM 4053, the sample of *S. angustmyceticus* JCM 4053; NRRL2666, the sample of *S. decoyicus* NRRL 2666. **b**, Theoretical fragmentation pattern of **1** and **2**. **c**, LC-HRMS/MS analysis of **1** and **2** produced by *S. angustmyceticus* JCM 4053. **d**, LC-HRMS/MS analysis of **1** and **2** produced by *S. decoyicus* NRRL 2666. **e**, The enzymatic reaction catalyzed by hydrolase LOG in *trans*-zeatin biosynthesis. LOG (Aceession no. AK071695) was utilized as a query sequence to the targeted identification of the angustmycin gene cluster.

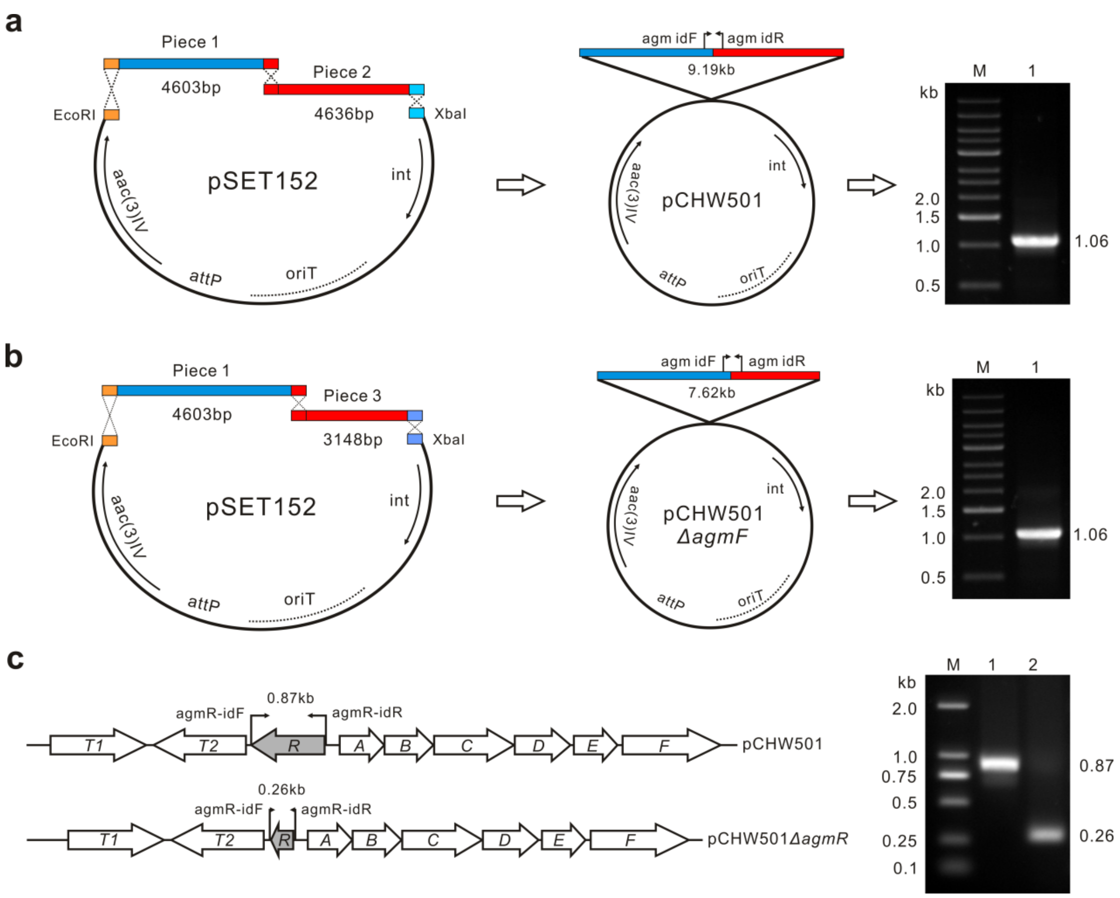

Supplementary Fig. 2. Cloning and heterologous expression of the *agm* gene cluster.

a, Diagrammatic sketch for the construction and identification of pCHW501. Right column, PCR identification of pCHW501, M, 1 kb ladder (Tsingke Biotech); 1, PCR product using pCHW501 as template. **b**, Diagrammatic sketch for the construction and identification of pCHW501*ΔagmF*. Right column, PCR identification of pCHW501*ΔagmF*, M, 1 kb ladder (Tsingke Biotech); 1, PCR product using pCHW501*ΔagmF* as template. **c**, In frame deletion of *agmR* in pCHW501 by Gibson Assembly method. Left column, schematic illustration for targeted *agmR* mutation. Right column, PCR identification of pCHW501*ΔagmR*. M, DL2000 ladder (Tsingke Biotech); 1, PCR product using pCHW501 as template; 2, PCR product using pCHW501*ΔagmR* as template. Each experiment was repeated independently with similar results for three times. Source data are provided as a Source Data file.

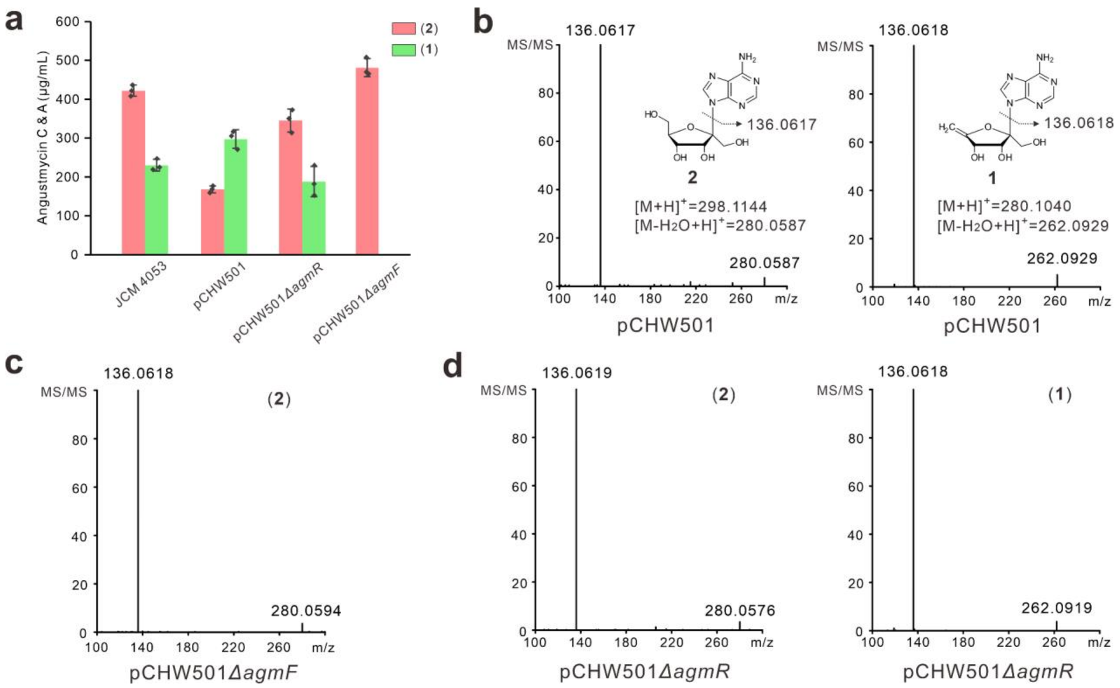

Supplementary Fig. 3. LC-HRMS/MS analysis of pCHW501 and its variants.
a, The production of **1** and **2** by related strains, including *S. angustmyceticus* JCM 4053, M1154::pCHW501, M1154::pCHW501*ΔagmR* and M1154::pCHW501*ΔagmF*. the error bars represent the ± s.d. from three different experiments. Source data are provided as a Source Data file. **b**, LC-HRMS/MS analysis of **1** and **2** produced by *S. coelicolor* M1154::pCHW501. **c**, LC-HRMS/MS analysis of **2** produced by *S. coelicolor* M1154::pCHW501*ΔagmF*. **d**, LC-HRMS/MS analysis of **1** and **2** produced by *S. coelicolor* M1154::pCHW501*ΔagmR*.

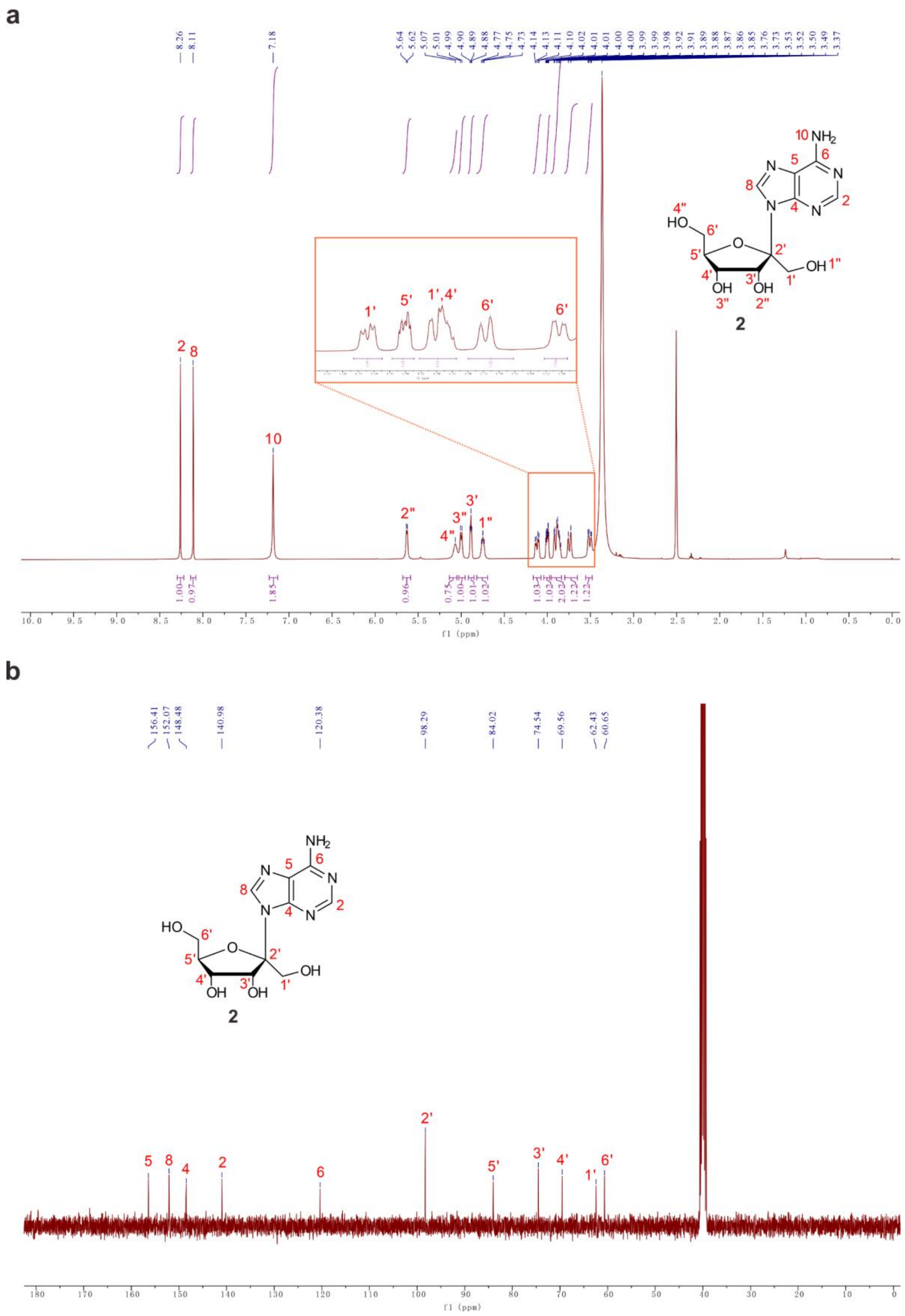

Supplementary Fig. 4. ^{1}H NMR and ^{13}C NMR data of 2.

a, ^{1}H NMR (400 MHz, DMSO-d6) δ 8.26 (1H, s), 8.11 (1H, s), 7.18 (2H, s), 5.63 (1H, d, J=4.7 Hz), 5.07 (1H, brs), 5.00 (1H, d, J=6.8 Hz), 4.89 (1H, t, J=4.1 Hz), 4.75(1H, t, J=5.1 Hz), 4.12 (1H, dd, J= 12.2, 5.1 Hz), 4.00 (1H, ddd, J=8.2, 4.0, 2.5 Hz), 3.89 (1H, m), 3.87 (1H, m), 3.74 (1H, d, J=12.1 Hz), 3.51 (1H, dd, J=12.1, 4.0 Hz). **b**, ^{13}C NMR (400 MHz, DMSO-d6), δ 156.4, 152.1, 148.5, 141.0, 120.4, 98.3, 84.0, 74.5, 69.6, 62.4, 60.7.

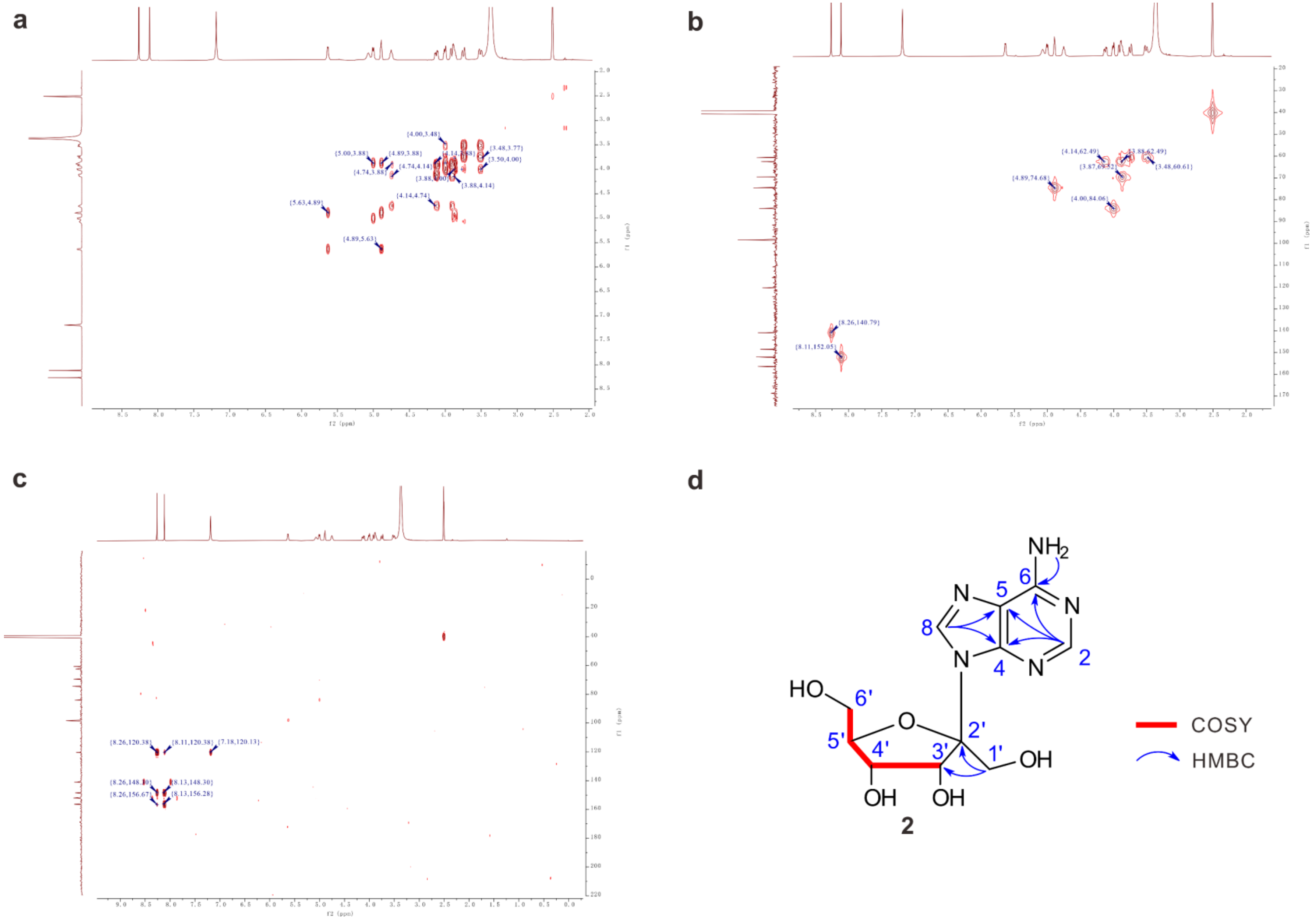
a
b
c
d
NH2
N
N
N
N
HO
O
OH
OH
OH
COSY
HMBC
2

Supplementary Fig. 5. 2D NMR analysis of 2.

a, ^1H-^1H COSY NMR analysis of **2**. **b**, HMQC NMR analysis of **2**. **c**, HMBC NMR analysis of **2**. **d**, 2D NMR analysis (based on COSY and HMBC) of **2**.

a

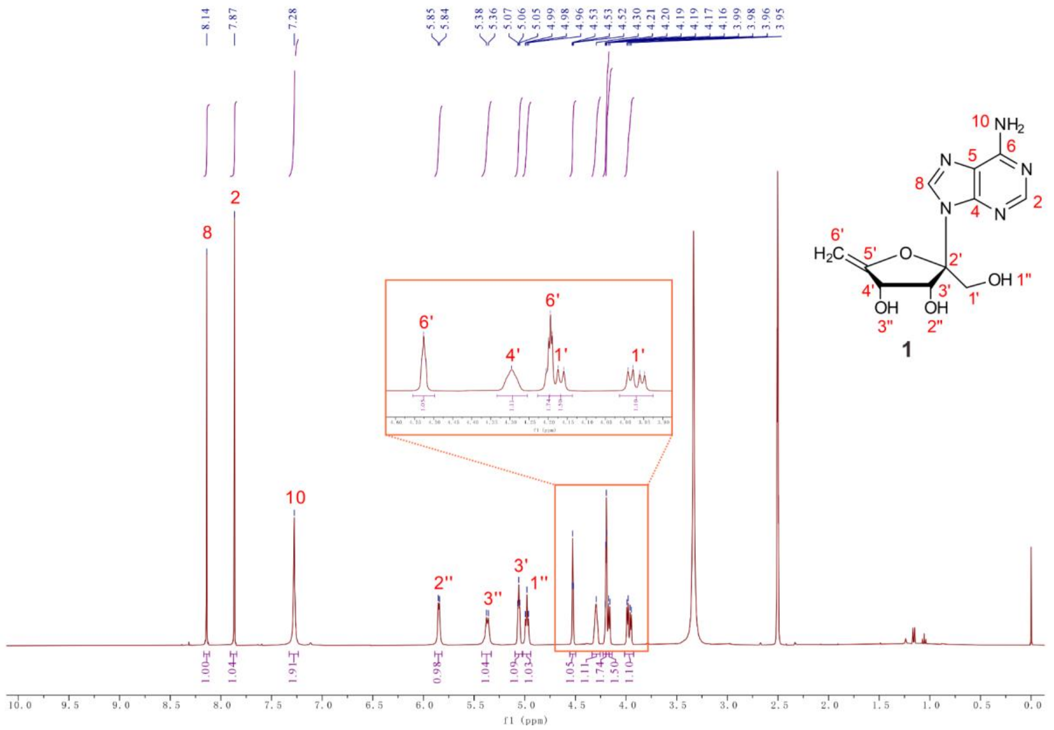

b

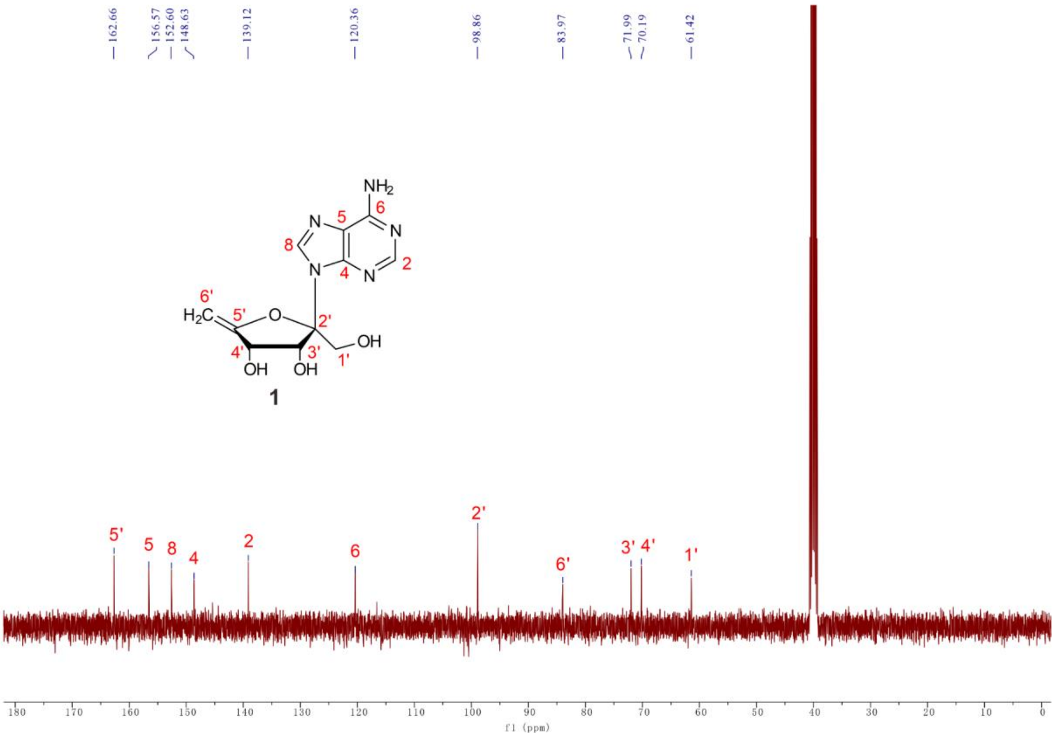

Supplementary Fig. 6. ^{1}H NMR and ^{13}C NMR data of 1.

a, ^{1}H NMR (400 MHz, DMSO-d6) δ 8.14 (1H, s), 7.78 (1H, s), 7.28 (2H, s), 5.85 (1H, d, J=4.6 Hz), 5.36 (1H, d, J=8.0 Hz), 5.06 (1H, t, J = 4.2 Hz), 4.98 (1H, t, J=6.0 Hz), 4.53(1H, brs), 4.3 (1H, brs), 4.19 (1H, t, J = 2.2 Hz), 4.18 (1H, dd, J=12.2, 5.1 Hz). **b**, ^{13}C NMR (400 MHz, DMSO-d6), δ 162.7, 156.6, 152.6, 148.6, 139.1, 120.4, 98.9, 84.0, 72.0, 70.2, 61.4.

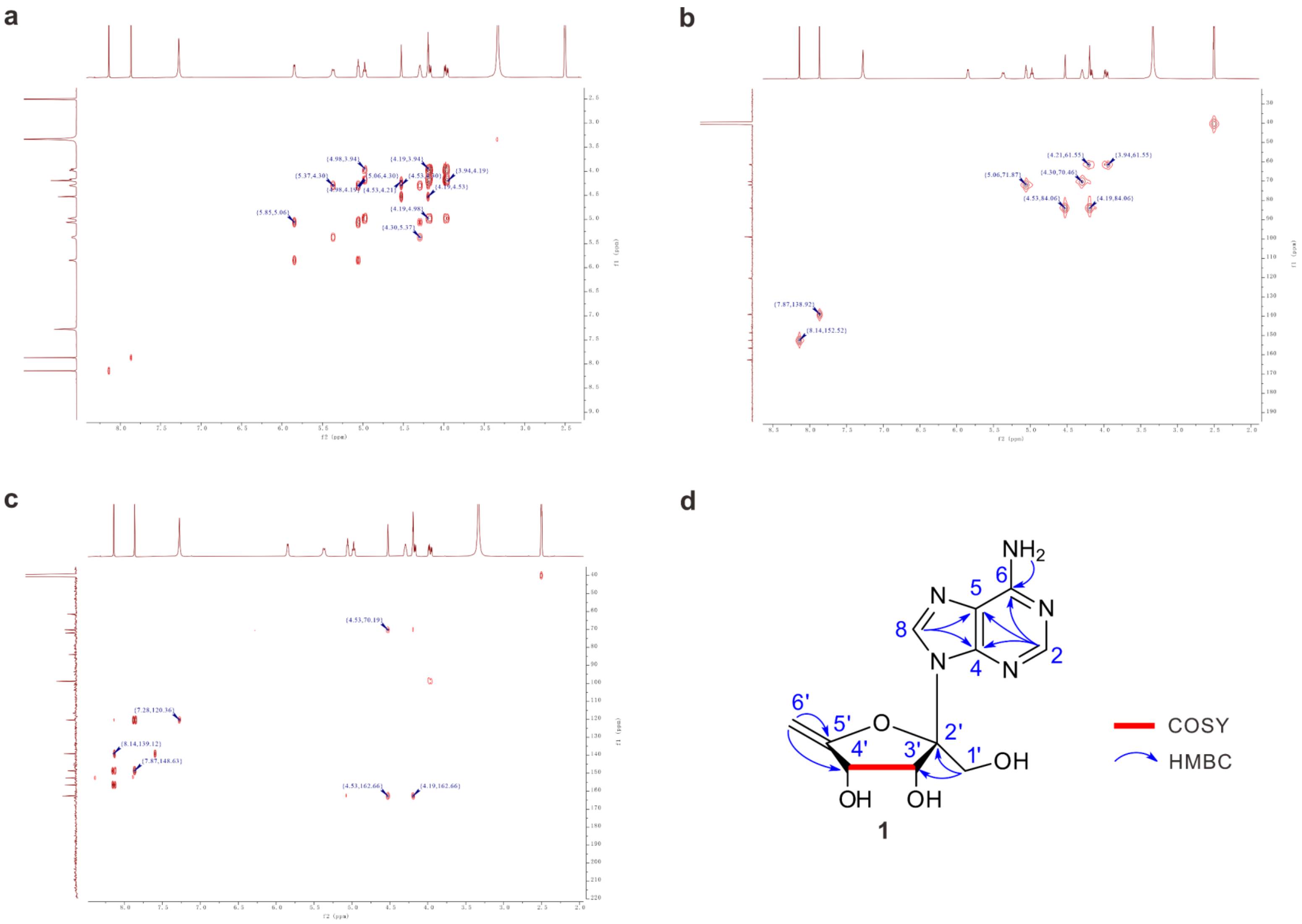
a
b
c
d
NH2
N
N
N
N
O
OH
OH
OH
1
COSY
HMBC

Supplementary Fig. 7. 2D NMR analysis of 1.

a, ^{1}H-^{1}H COSY NMR analysis of **1**. **b**, HMQC NMR analysis of **1**. **c**, HMBC NMR analysis of **1**. **d**, 2D NMR analysis (based on COSY and HMBC) of **1**.

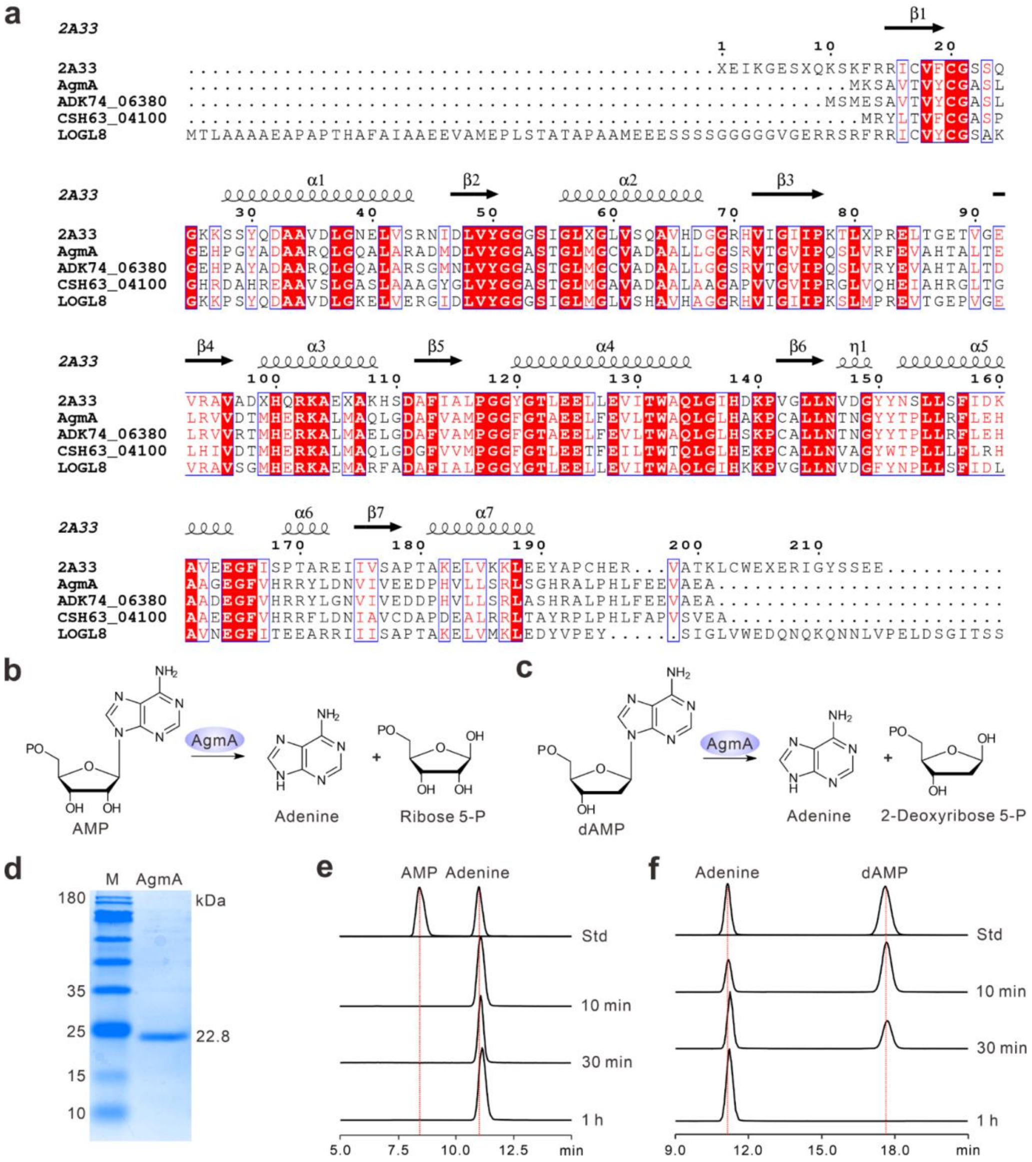

Supplementary Fig. 8. Functional analysis of AgmA as a phosphoribohydrolase for adenine supply.

a, Sequence alignment of AgmA with its homologs using ESPript 3.0[2]. Proteins include 2A33 (PDB: 2A33_A, https://www.rcsb.org/structure/2A33) from *Arabidopsis thaliana*, ADK74_06380 (GenBank: KOG49021) from *Streptomyces decoyicus*, CSH63_04100 (GenBank: AYF26661) from *Micromonospora tulbaghiae*, and LOGL8 (GenBank: XP_015639529) from *Oryza sativa* Japonica Group, and secondary structure of 2A33 is displayed on the top. **b**, Scheme of the AgmA-catalyzed reaction with AMP as substrate. **c**, Scheme of the AgmA-catalyzed reaction with dAMP as substrate. **d**, SDS-PAGE analysis of the protein AgmA (22.8 kDa). Source data are provided as a Source Data file. Experiments were repeated independently with similar results for three times. **e**, HPLC analysis (λ=254 nm) of AgmA-catalyzed reaction under different time courses using AMP as substrate. Std, the authentic AMP and adenine standard; 10 min, 30 min and 1 h indicate AgmA reaction conducted in 10 min, 30 min and 1 h, respectively. **f**, HPLC analysis (λ=254 nm) of AgmA-catalyzed reaction under different time courses using dAMP as substrate. Std, the authentic dAMP and adenine standard; 10 min, 30 min and 1 h indicate AgmA reaction conducted in 10 min, 30 min and 1 h, respectively.

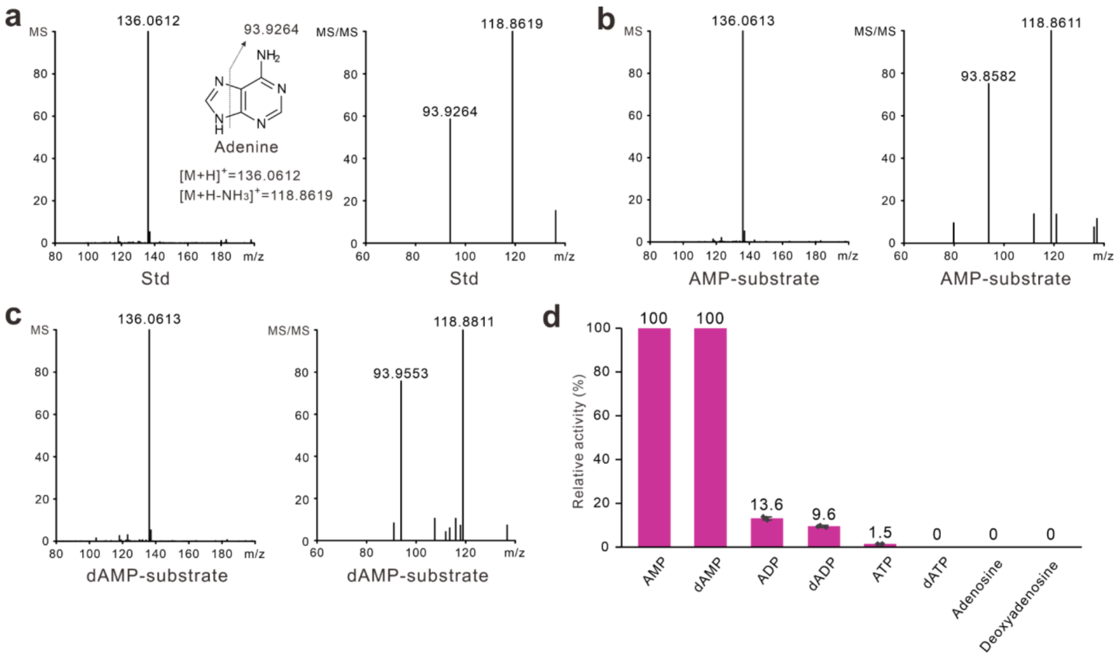

Supplementary Fig. 9. LC-HRMS analysis of AgmA catalyzed reaction and the substrate specificity of AgmA.

a, LC-HRMS analysis of the authentic adenine standard. **b**, LC-HRMS analysis of the target product (adenine) from the AgmA-catalyzed reaction using AMP as substrate. **c**, LC-HRMS/MS analysis of the target product (adenine) from the AgmA-catalyzed reaction using dAMP as substrate. **d**, Relative activity of AgmA in 2 h against the different substrates based on the production of adenine, the error bars represent the ± s.d. from three different experiments. Source data are provided as a Source Data file.

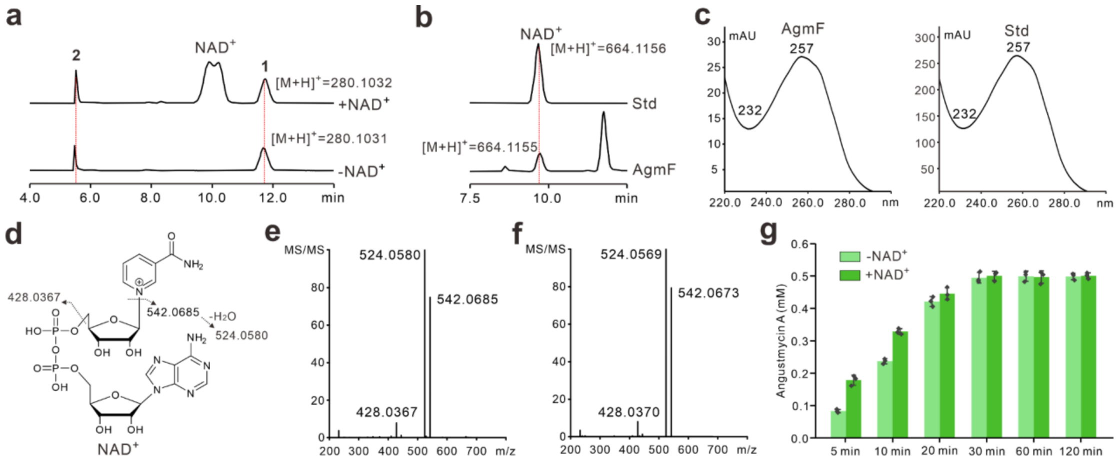

Supplementary Fig. 10. Characterization of the cofactor NAD$^+$ bound in AgmF. **a**, HPLC analysis (λ=254 nm) of AgmF-catalyzed reaction with or without cofactor NAD$^+$. +NAD$^+$, the AgmF reaction adding NAD$^+$; -NAD$^+$, AgmF reaction conducted without NAD$^+$. **b**, HPLC analysis (λ=254 nm) of the cofactor NAD$^+$ bound in AgmF. Std, the authentic NAD$^+$ standard; AgmF, the cofactor contained in the boiled AgmF. **c**, UV spectrum of NAD$^+$ standard and the corresponding cofactor detected in AgmF. **d**, Fragmentation pattern of the NAD$^+$ cofactor. **e**, LC-HRMS analysis of the authentic NAD$^+$ standard. **f**, LC-HRMS analysis of the counterpart cofactor from AgmF. **g**, Time-course experiment of AgmF reaction with or without NAD$^+$ (1 mM) added. The values of Y-coordinate represent **1** production (mM). the error bars represent the ± s.d. from three different experiments. *The exact mass of the NAD$^+$ (uncharged) cofactor is calculated as 663.1091 in this figure. Source data are provided as a Source Data file.

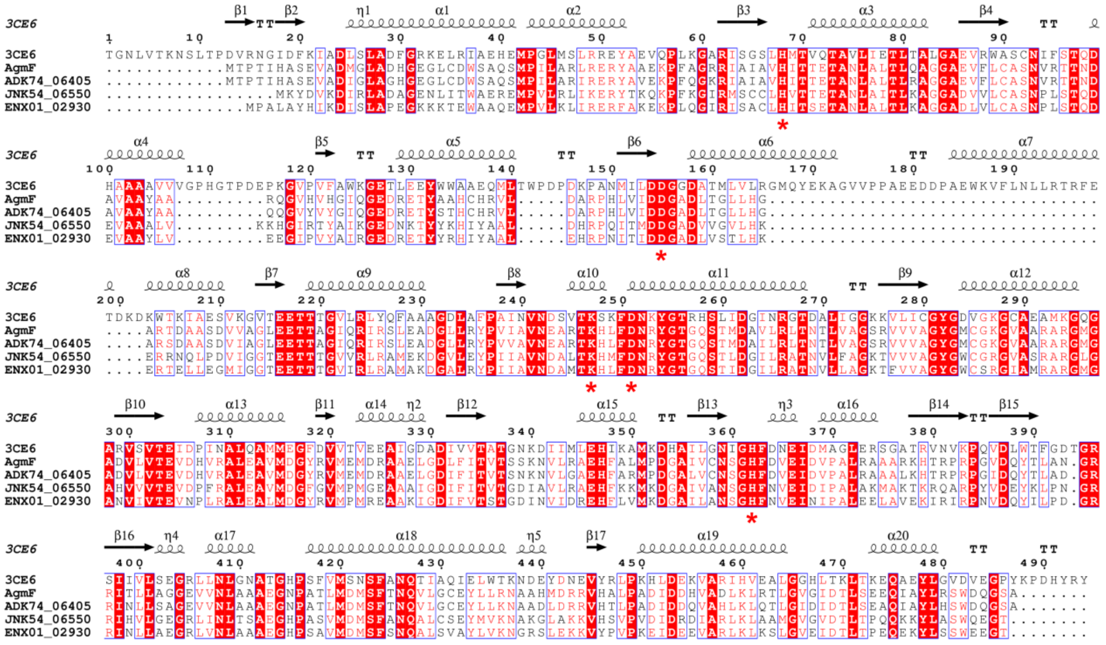

Supplementary Fig. 11. Bioinformatic analysis of AgmF with its homologs. Proteins include 3CE6 (PDB: 3CE6_A, https://www.rcsb.org/structure/3CE6) from *Mycobacterium tuberculosis* H37Rv, ADK74_06405 (GenBank: KOG49016) from *Streptomyces decoyicus*, JNK54_06550 (GenBank: MBL8023925) from *Elusimicrobia bacterium*, and ENX01_02930 (GenBank: HFU13397) from *Anaerolineae bacterium*. Secondary structure of 3CE6 is shown on the top, and the conserved active sites are highlighted with red asterisk.

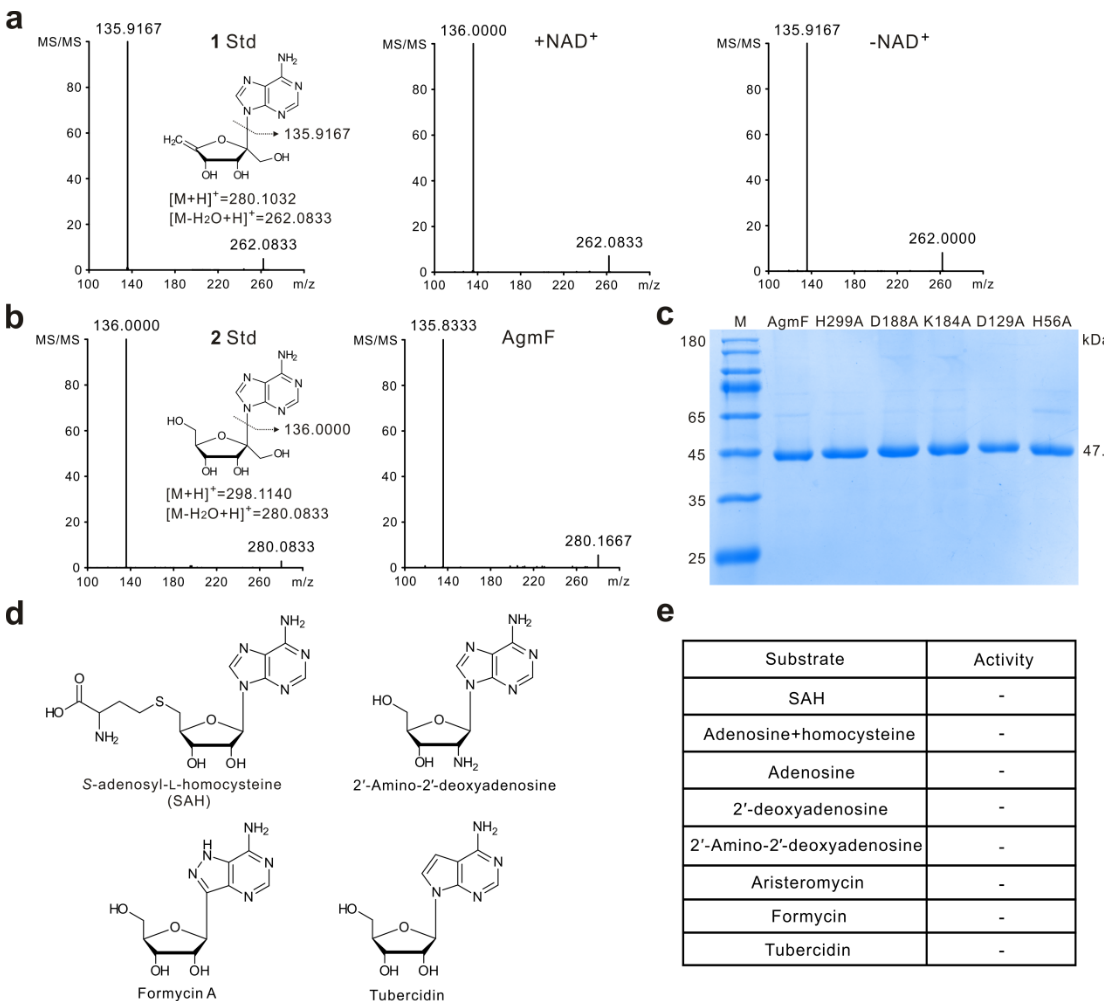

Substrate	Activity
SAH	-
Adenosine+homocysteine	-
Adenosine	-
2′-deoxyadenosine	-
2′-Amino-2′-deoxyadenosine	-
Aristeromycin	-
Formycin	-
Tubercidin	-

Supplementary Fig. 12. Functional analysis of AgmF as a 2 dehydratase.

a, LC-HRMS/MS analysis of the authentic **1** standard, AgmF reaction (with or without NAD^+ added). **b**, LC-HRMS/MS analysis of the authentic **1** standard and AgmF reverse-reaction. **c**, SDS-PAGE analysis of AgmF and its variants (47.5 kDa). Source data are provided as a Source Data file. Experiments were repeated independently with similar results for three times. **d**, Chemical structure of the potential substrates for AgmF. **e**, The AgmF reactions against diverse substrates. The "-" denotes "no activity".

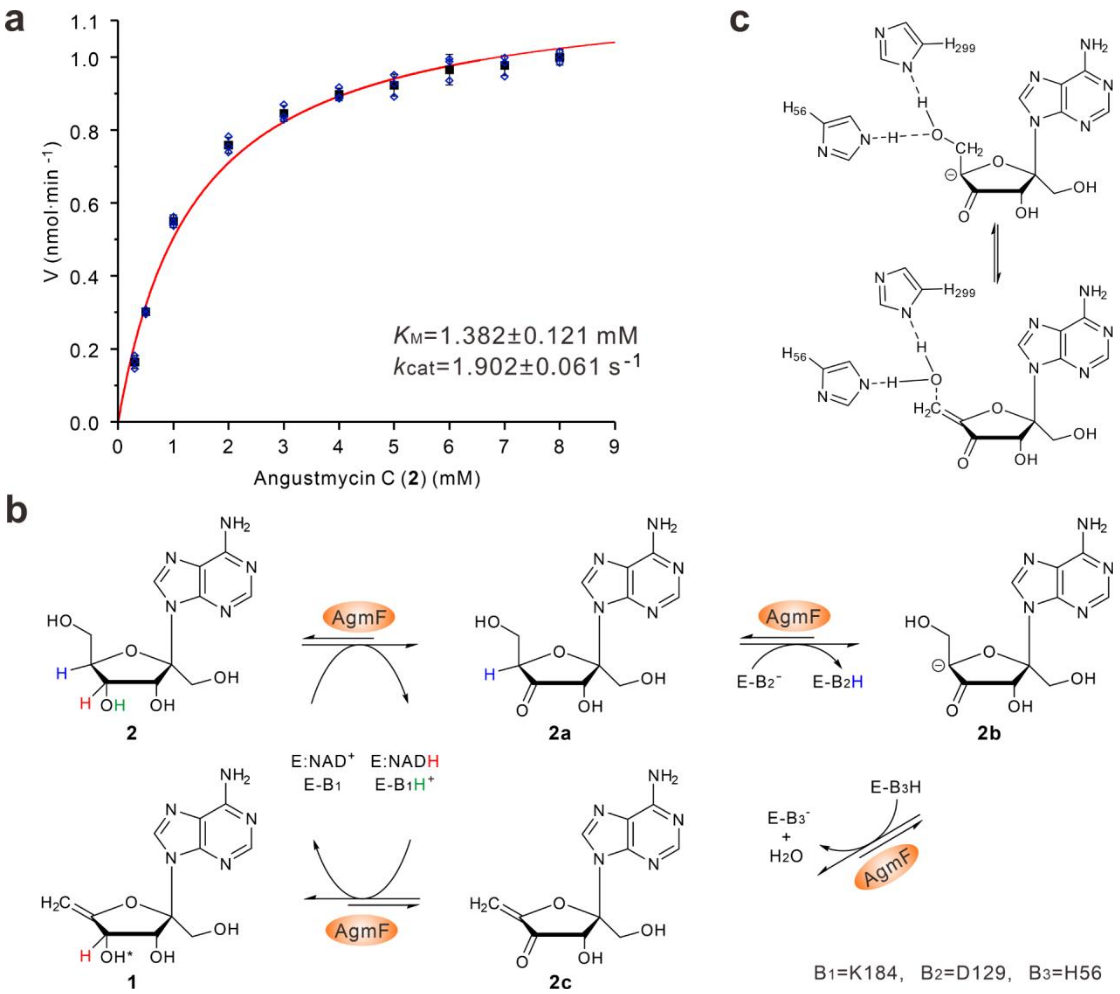

Supplementary Fig. 13. Kinetic analysis of AgmF angainst 2 and concise enzymatic mechanism for the AgmF-catalyzed reaction.

a, Kinetic analysis of AgmF for **2**. The reactions were performed with **2** ranging from 0.3 to 8 mM at 30°C in a total volume of 20 μL that contained 2 ng AgmF in 25 min. K_M and k_{cat} values represent the mean ± s.d. of three independent replicates. Source data are provided as a Source Data file. **b**, Proposed cofactor-cycling mechanism for the AgmF-catalyzed reaction. **c**, H56 and H299 are speculated to form hydrogen bonds with O6′ of **2** and participate in the reaction for the removal of hydroxyl group.

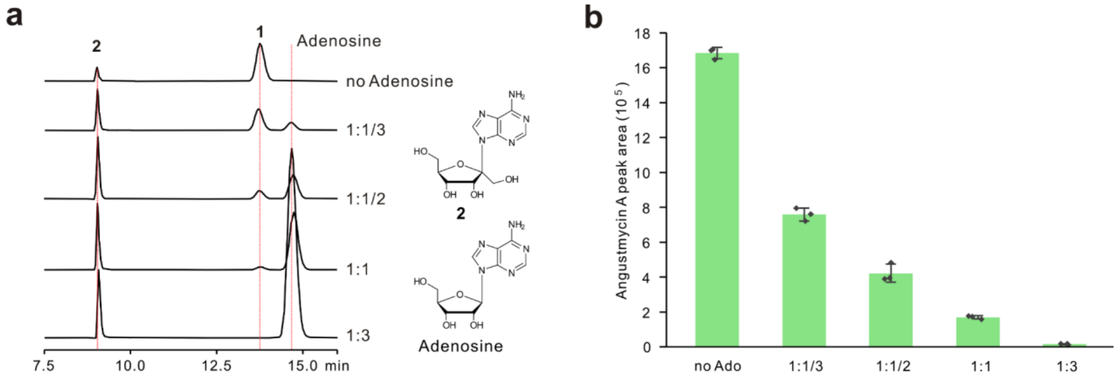

Supplementary Fig. 14. The competitive inhibition of AgmF by adenosine.

a, The adenosine competitive inhibition to **2** dehydratase activity (λ=254 nm). No Adenosine, AgmF-catalyzed reaction with 1 mM **2** added; 1:1/3, AgmF-catalyzed reaction with 1 mM **2** and 0.33 mM adenosine added; 1:1/2, reaction with 1 mM **2** and 0.5 mM adenosine added; 1:1, reaction with 1 mM **2** and 1 mM adenosine added; 1:3, reaction with 1 mM **2** and 3 mM adenosine added. **b**, The production of **1** in the adenosine competitive inhibition reaction. The **1** production was indicated by the peak area of **1** based on the detection wavelength of 254 nm, on account of the range of the standard curve. The error bars represent the ± s.d. from three different experiments. Source data are provided as a Source Data file.

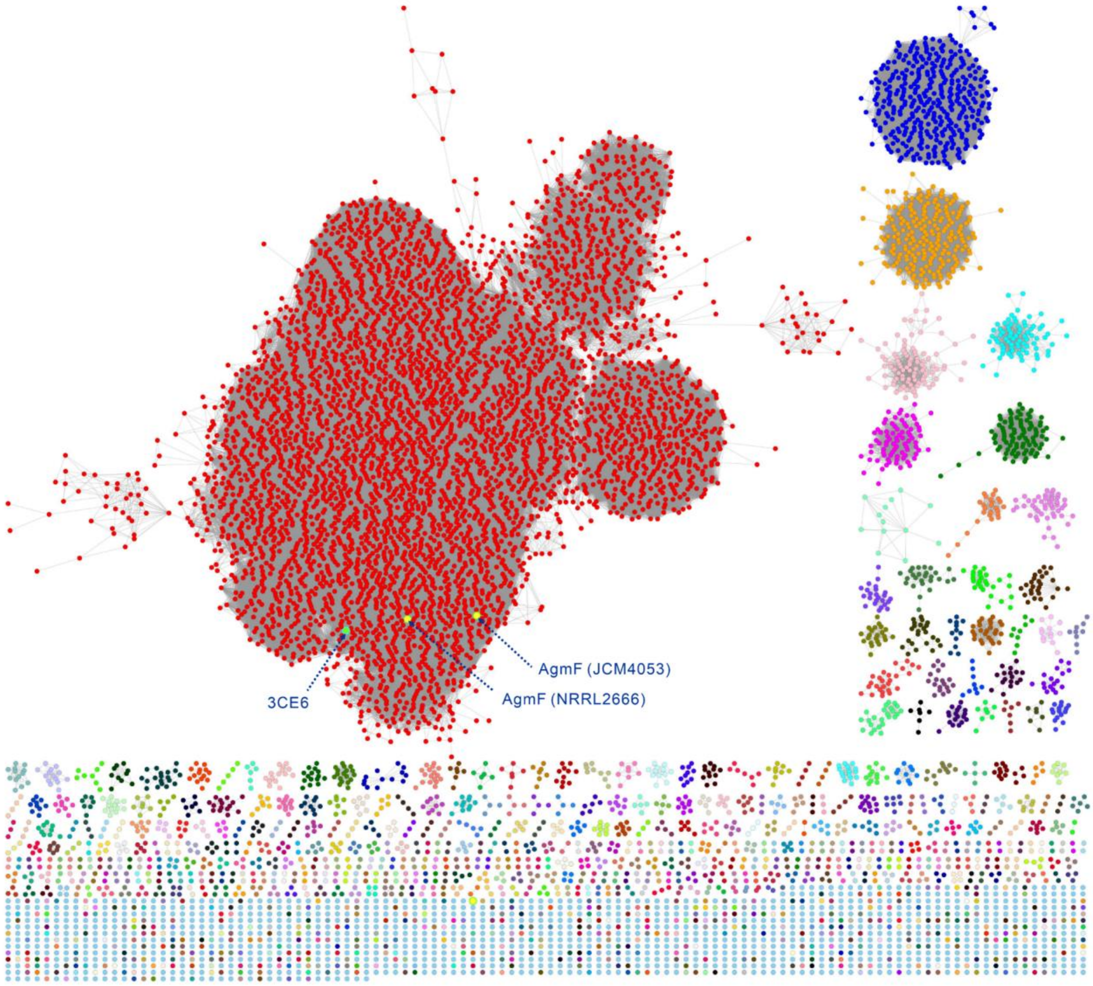

Supplementary Fig. 15. The colored-SSN analysis of the SAHH-type proteins. SSN analysis of the SAHH (SAH hydrolase) family proteins in microbial systems. The Pfam family number is PF05221, and the SSN was generated using the UniRef90 database with alignment score of 200. AgmF and the structural model protein 3CE6 belong to a distinct SAHH family (red cluster) which is away from other types of SAHHs[3,4]. The colored-SSN analysis was generated by Cytoscape (v3.8.2).

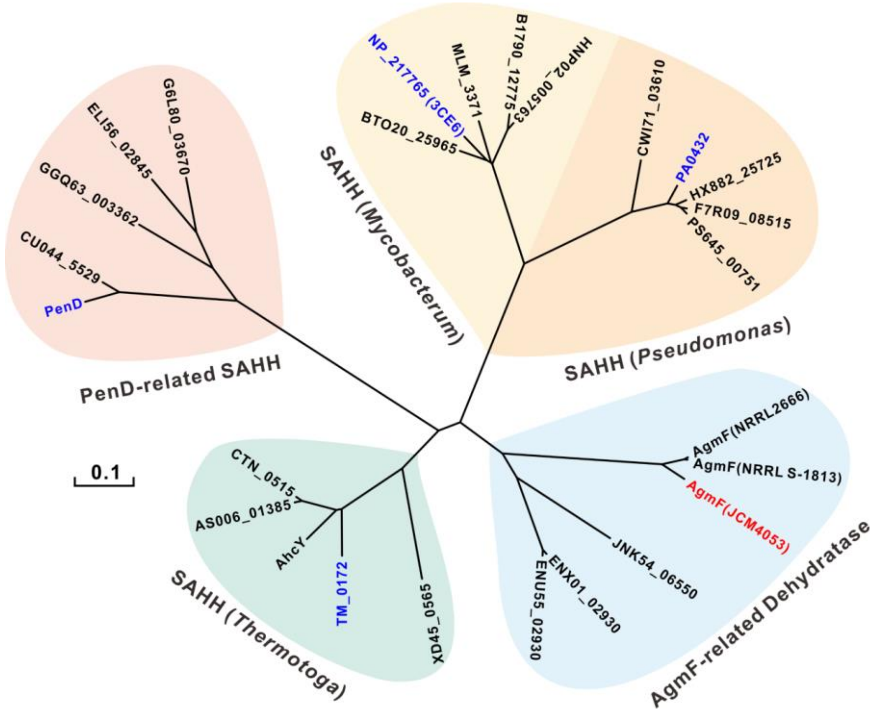

Supplementary Fig. 16. Phylogenetic analysis of AgmF against other SAHHs.
The AgmF-related dehydratase cluster includes AgmF (NRRL2666) (ADK74_06405, GenBank: KOG49016) from *Streptomyces decoyicus*, AgmF (NRRL S-1813) (GenBank: WP_030986632) from *Streptomyces* sp. NRRL S-1813, JNK54_06550 (GenBank: MBL8023925) from *Elusimicrobia bacterium*, ENX01_02930 (GenBank: HFU13397) from *Anaerolineae bacterium*, and ENU55_02930 (GenBank: HGQ07556) from *Chloroflexi bacterium*; The SAHH (SAH hydrolase, *Thermotoga*) cluster contains TM_0172 (GenBank: AAD35265) from *Thermotoga maritima* MSB8, CTN_0515 (GenBank: ACM22691) from *Thermotoga neapolitana* DSM 4359, AS006_01385 (GenBank: PLV57550) from *Thermotoga* sp. SG1. AhcY (GenBank: CAI44380) from *Thermotoga* sp. KOL6, and XD45_0565 (GenBank: KUK03340) from *Thermotoga* sp. 50_64; The PenD-related SAHH cluster includes PenD (GenBank: AKA87337) from *Streptomyces antibioticus*, CU044_5529 (GenBank: RYJ23224) from *Streptomyces* sp. L-9-10, GGQ63_003362 (GenBank: MBB5754281) from *Prosthecomicrobium pneumaticum*, and ELI56_02845 (GenBank: TAT77216) from *Rhizobium leguminosarum*. The SAHH (Mycobacterum) cluster contains NP_217765 (GenBank: P9WGV3) from *Mycobacterium tuberculosis* H37Rv, BTO20_25965 (GenBank: ART71536) from *Mycobacterium dioxanotrophicus*, MLM_3371 (GenBank: ATA29380) from *Mycobacterium lepraemurium*, B1790_12775 (GenBank: OPX10083) from *Mycobacterium* sp. AT1, and HNP02_005763 (GenBank: MBB5165781) from *Mycobacterium* sp. AZCC_0083; The SAHH (*Pseudomonoas*) cluster contains PA0432 (GenBank: Q9I685) from *Pseudomonas aeruginosa* PAO1, CWI71_03610 (GenBank: RUO62532) from *Pseudidiomarina insulisalsae*, HX882_25725 (GenBank: NWB99297) from *Pseudomonas gingeri*, F7R09_08515 (GenBank: KAB0497552) from *Pseudomonas vancouverensis*, and PS645_00751 (GenBank: VVM51021) from *Pseudomonas fluorescens*. SAHH, SAH hydrolase.

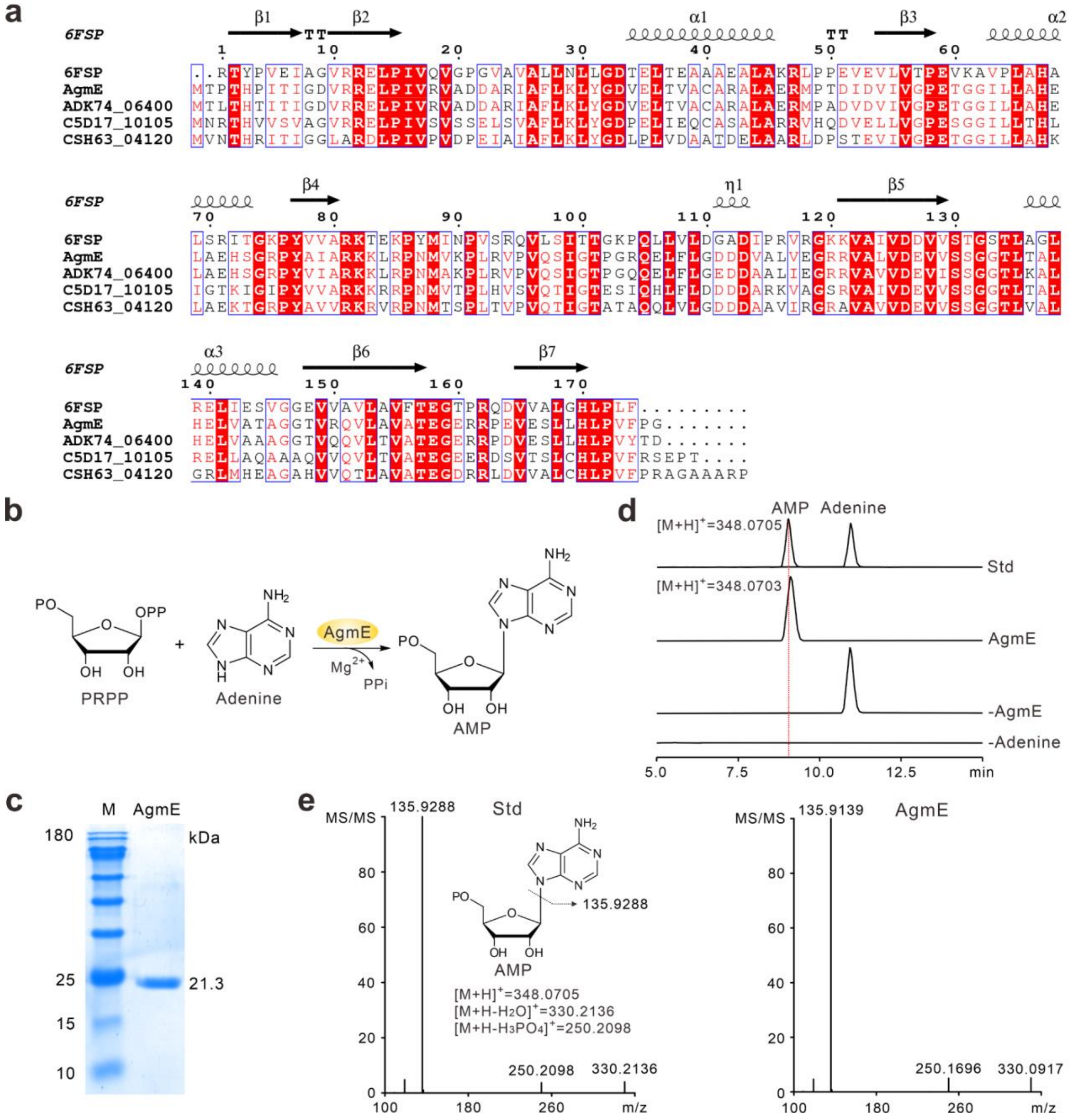

Supplementary Fig. 17. Functional analysis of AgmE as an adenine phosphoallulosyltransferase.

a, Bioinformatic analysis of AgmE with its homologs. Proteins contain 6FSP (PDB: 6FSP_A, https://www.rcsb.org/structure/6FSP) from *Thermus thermophiles*, ADK74_06400 (GenBank: KOG49015) from *Streptomyces decoyicus*, C5D17_10105 (GenBank: PPH21182) from *Rathayibacter toxicus,* and CSH63_04120 (GenBank: AYF26665) from *Micromonospora tulbaghiae*, and secondary structure of 6FSP is displayed on the top. **b**, Scheme of the AgmE-catalyzed reaction with adenine and PRPP as substrate. **c**, SDS-PAGE analysis of the protein AgmE (21.3 kDa). Source data are provided as a Source Data file. Experiments were repeated independently with similar results for three times. **d**, HPLC analysis (λ=254 nm) of AgmE-catalyzed reaction with adenine and PRPP as substrate. Std, the authentic AMP and adenine standards; AgmE, AgmE-catalyzed reaction; -AgmE, AgmE reaction without AgmE added; -Adenine, AgmE reaction without adenine added. **e**, LC-HRMS/MS analysis of the authentic AMP standard and target product (AMP) from the AgmE-catalyzed reaction using adenine and PRPP as substrate.

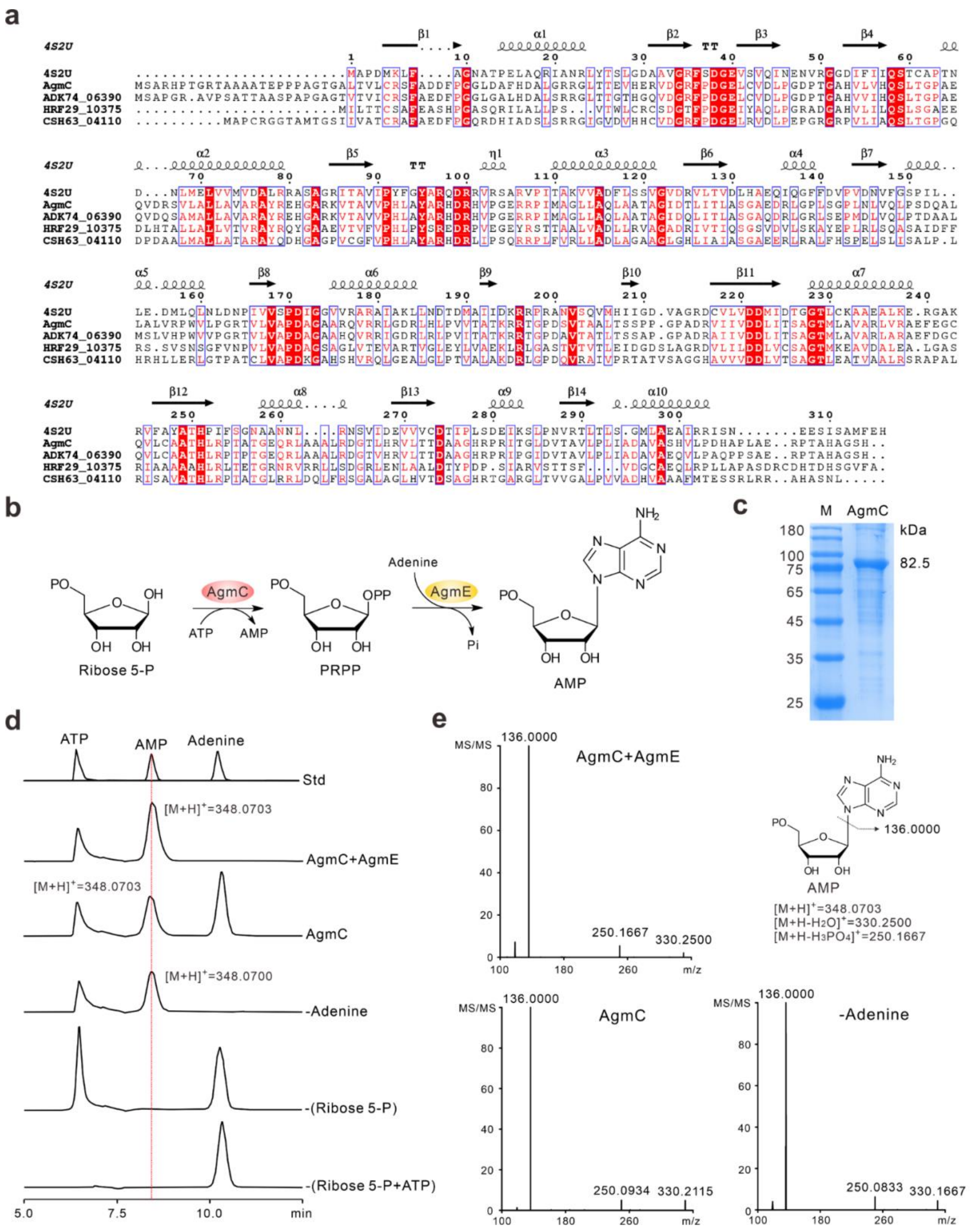

Supplementary Fig. 18. Functional analysis of AgmC as a Ribose 5-P pyrophosphokinase.

a, Bioinformatic analysis of AgmC with its homologs. The following proteins are included: 4S2U (PDB: 4S2U_A, https://www.rcsb.org/structure/4S2U) from *Escherichia coli*; ADK74_06390 (GenBank: KOG49014) from *Streptomyces decoyicus*; HRF29_10375 (GenBank: NRD09337) from *Rathayibacter agropyri*; CSH63_04110 (GenBank: AYF26663) from *Micromonospora tulbaghiae*, and secondary structure of 6NFE is displayed on the top. **b**, Scheme of the AgmC-catalyzed reaction with ribose 5-phosphate and ATP as substrate. **c**, SDS-PAGE analysis of the protein AgmC with a fusion maltose binding protein (MBP)

tag (82.5 kDa). Source data are provided as a Source Data file. Experiments were repeated independently with similar results for three times. **d**, HPLC analysis (λ=254 nm) of AgmC and AgmE combined reaction with ribose 5-phosphate, adenine, and ATP as substrate. Std, the authentic ATP, AMP, and adenine standards; AgmC+AgmE, AgmC and AgmE coupled reaction; AgmC, AgmC reaction; -Adenine, coupled reaction without adenine added; -(Ribose 5-P), coupled reaction without ribose 5-phosephate added; -(Ribose 5-P+ATP), coupled reaction without ribose 5-phosephate and ATP added. **e**, LC-HRMS/MS analysis of the target product (AMP) from the AgmC and AgmE coupled reaction, AgmC reaction, and AgmC and AgmE coupled reaction without adding of Adenine.

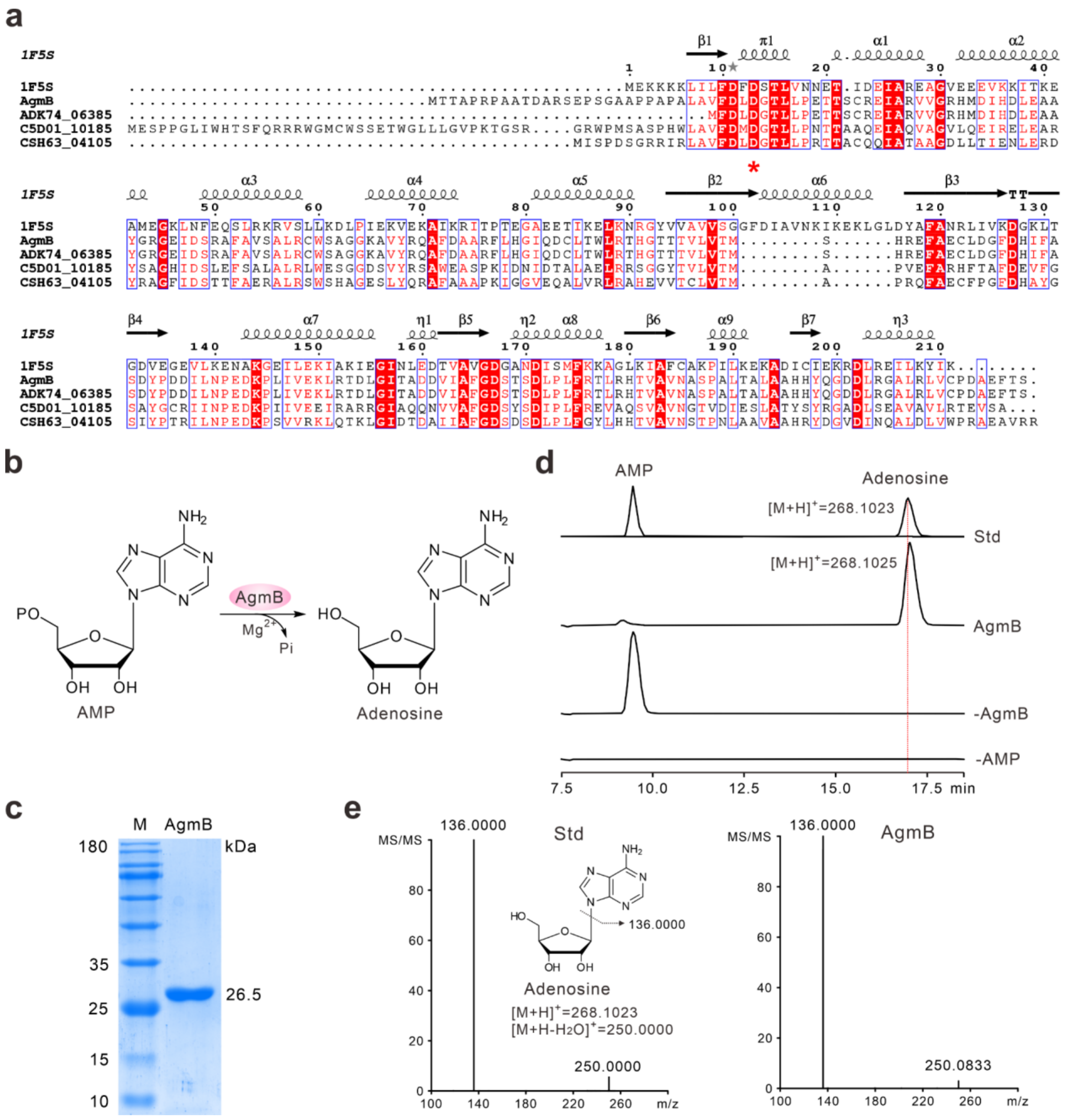

Supplementary Fig. 19. Functional analysis of AgmB as a HAD-like hydrolase.
a, Bioinformatic analysis of AgmB with its homologs. The following proteins are included: 1F5S (PDB: 1F5S_A, https://www.rcsb.org/structure/1F5S) from *Methanocaldococcus jannaschii*; ADK74_06385 (GenBank: KOG49022) from *Streptomyces decoyicus*; C5D01_10185 (GenBank: PPH67114) from *Rathayibacter toxicus*; CSH63_04105 (GenBank: AYF26662) from *Micromonospora tulbaghiae*. The conserved active sites are highlighted with red asterisk, and secondary structure of 1F5S is displayed on the top. **b**, Scheme of the AgmB-catalyzed reaction with AMP as substrate. **c**, SDS-PAGE analysis of the protein AgmB (26.5 kDa). Source data are provided as a Source Data file. Experiments were repeated independently with similar results for three times. **d**, HPLC analysis (λ=254 nm) of AgmB-catalyzed reaction with AMP as substrate. Std, the authentic AMP and adenosine standards; AgmB, AgmB-catalyzed reaction; -AgmB, AgmB reaction without AgmB added; -AMP, AgmB reaction without AMP added. **e**, LC-HRMS/MS analysis of the authentic adenosine standard and target product (adenosine) from the AgmB-catalyzed reaction using AMP as substrate.

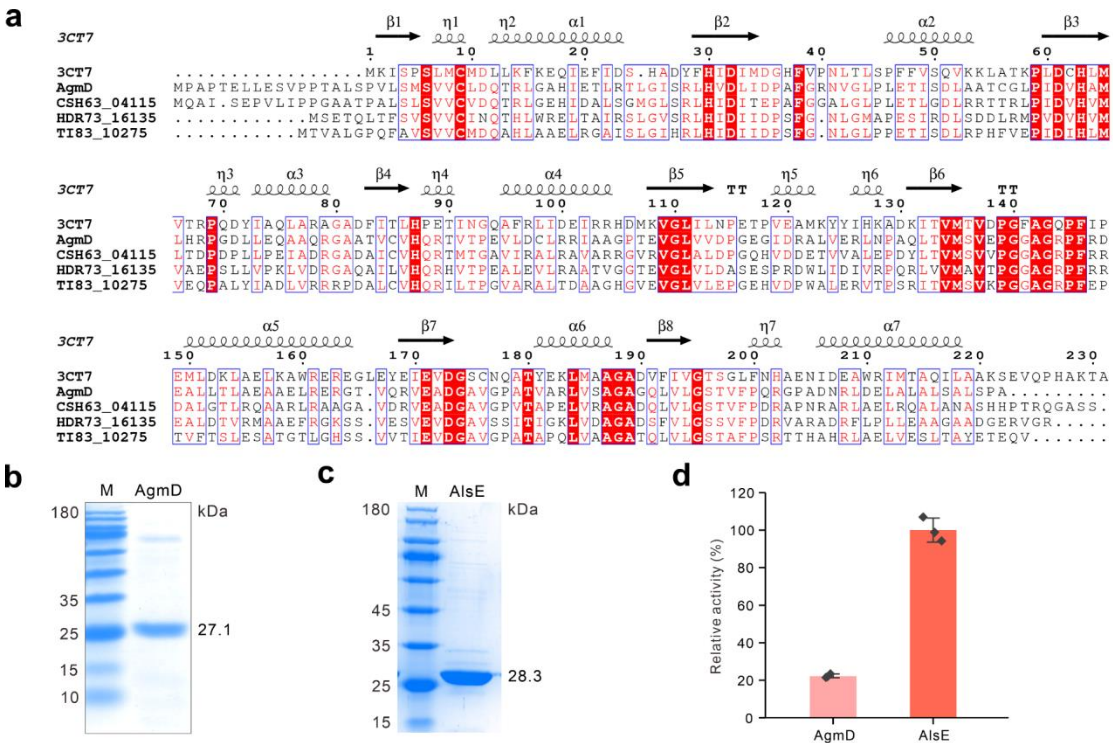

Supplementary Fig. 20. Bioinformatics analysis of AgmD with its homologs.

a, Bioinformatic analysis of AgmD with its homologs. The following proteins are included: 3CT7 (PDB: 3CT7_A, https://www.rcsb.org/structure/3CT7) from *Escherichia Coli* K-12; CSH63_04115 (GenBank: AYF26664) from *Micromonospora tulbaghiae*; HDR73_16135 (GenBank: MBD5383323) from *Clavibacter* sp.; TI83_10275 (GenBank: AJM78207) from *Rathayibacter toxicus*, and secondary structure of 3CT7 (AlsE) is displayed on the top. **b**, SDS-PAGE analysis of the protein AgmD (27.1 kDa). **c**, SDS-PAGE analysis of the protein AlsE (28.3 kDa). **d**, the relative activity comparison between AgmD and AlsE, one-pot reactions of (AlsE, AgmC, A, E, B) and (AgmD, C, A, E, B) were compared, and the average **2** production of AlsE-participated reaction was counted as 100 percent to calculate the relative activity of AgmD-participated reaction. The error bars represent the ± s.d. from three different experiments. Experiments were repeated independently with similar results for three times. Source data underlying Supplementary Fig. 20b-d are provided as a Source Data file.

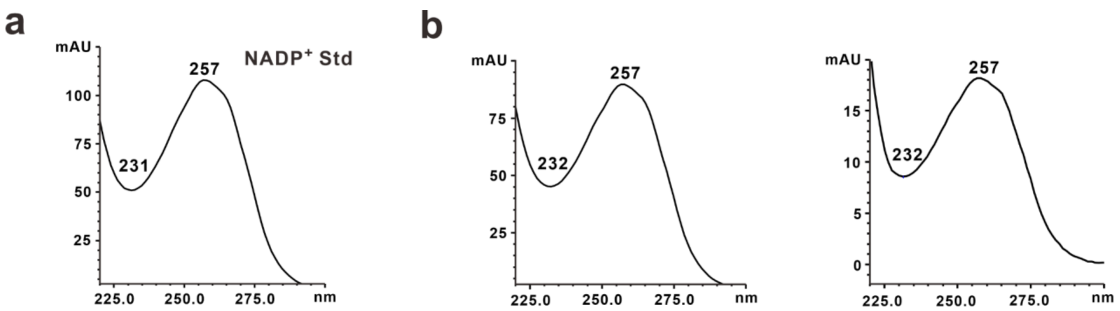

Supplementary Fig. 21. UV spectrum of NADP$^+$-related compounds.
a, UV spectrum of NADP$^+$ standard. **b**, UV spectrum of NADP$^+$-related compounds represented as "*" in Fig. 5C.

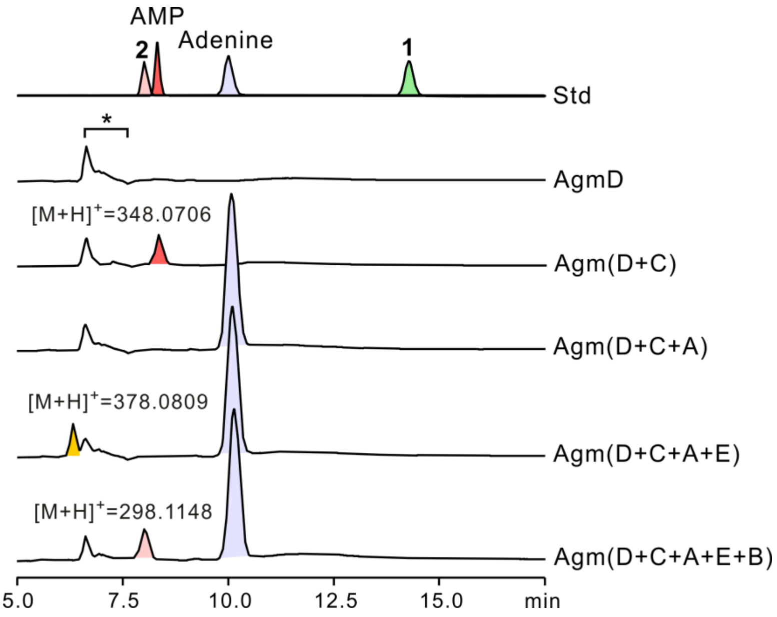

Supplementary Fig. 22. The stepwise reactions with AgmD as the starter enzyme. HPLC analysis (λ=254 nm) of the one-pot reaction mixtures of proteins responsible for angustmycin biosynthesis. Std, the authentic standards of **2**, AMP, adenine and **1**; AgmD, the reaction with only AgmD and related substrates, metal ion and cofactors added; Agm(D+C+A+E+B), the reaction with AgmD, AgmC, AgmA, AgmE, and AgmB complete five proteins added, accompanying with related substrates, metal ion, and cofactors. Other samples are correspondingly assigned. "*" represents NADP$^+$-related compounds.

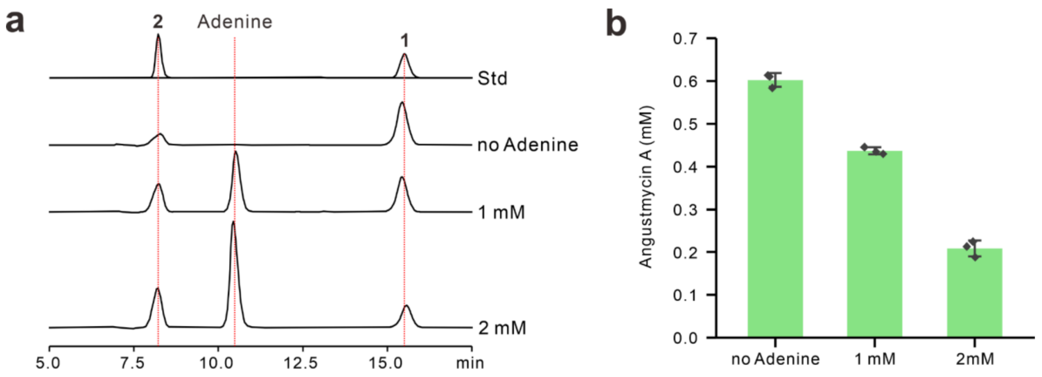

Supplementary Fig. 23. The competitive inhibition of adenine on AgmF.
a, HPLC analysis (λ=254 nm) of the adenine competitive inhibition to AgmF activity. Std, the authentic **2** and **1** standard; No Adenine, AgmF-catalyzed reaction with 1 mM **2** added; 1 mM, AgmF-catalyzed reaction with 1 mM **2** and 1 mM adenine added; 2 mM, reaction with 1 mM **2** and 2 mM adenine added. **b**, The production of **1** in the adenine competitive inhibition reaction. The **1** production was indicated by the molar concentration of **1** based on the detection wavelength of 254 nm. The error bars represent the ± s.d. from three different experiments. Source data are provided as a Source Data file.

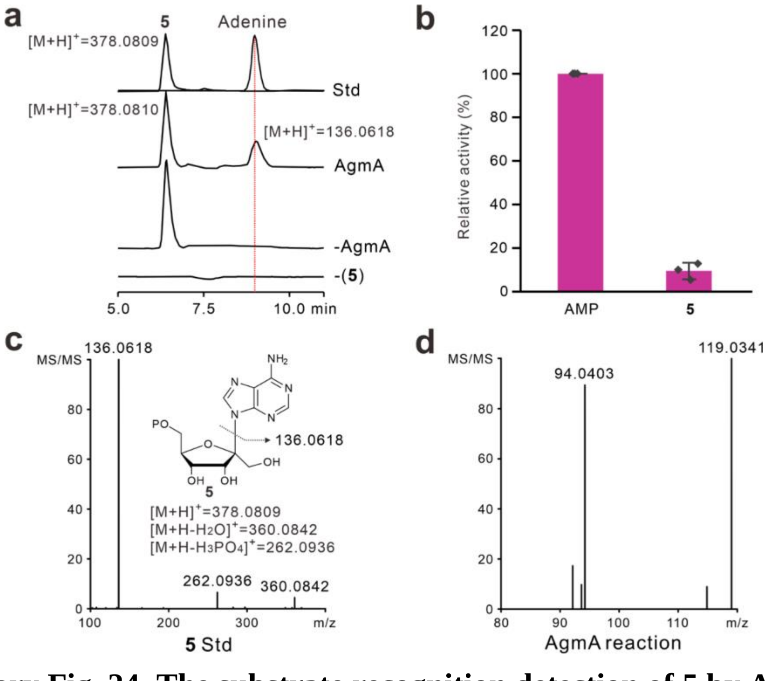

Supplementary Fig. 24. The substrate recognition detection of 5 by AgmA.
a, HPLC analysis (λ=254 nm) of the AgmA reaction to recognize **5**. Std, the authentic **5** and adenine standard; AgmA, AgmA-catalyzed reaction with **5** as substrate; -AgmA, AgmA reaction without AgmA added; -(**5**), AgmA reaction without **5** added. **b**, Relative activity of AgmA in 2 h against AMP and **5**, based on the production of adenine. The error bars represent the ± s.d. from three different experiments. Source data are provided as a Source Data file. **c**, LC-HRMS/MS analysis of the **5** standard. **d**, LC-HRMS/MS analysis of the target product (adenine) from the AgmA-catalyzed reaction using **5** as substrate.

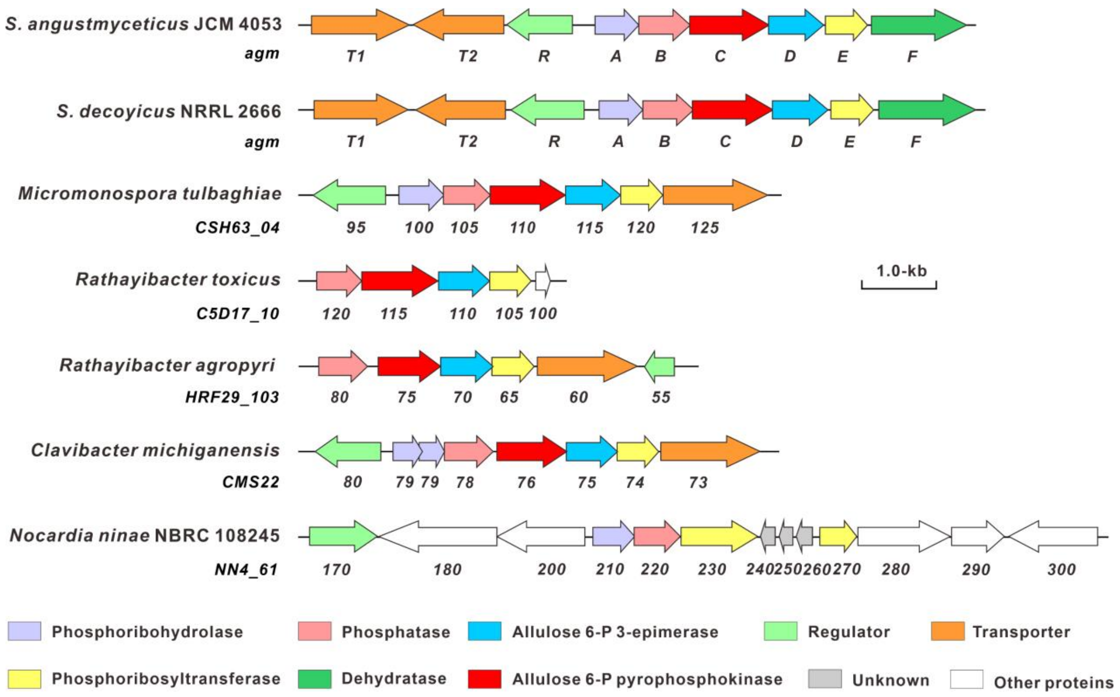

Supplementary Fig. 25. Target-directed genome mining of the gene clusters for the potential angustmycin related nucleoside cytokinins.

The potential gene clusters of the angustmycin related cytokinins were obtained using the AgmE as query sequence, and the proposed gene functions with highlighted colors were listed at the bottom.

Supplementary Table 1. NMR data of Angustmycin C in DMSO, 400MHz.

NO.	^{1}H (δ, multi)	^{13}C (δ)
2	8.26 s	141.0
4		148.5
5		156.4
6		120.4
8	8.11 s	152.1
1′	4.12 dd, 12.2, 5.1 Hz; 3.88 m	62.4
2′		98.3
3′	4.89 t, 4.1 Hz	74.5
4′	3.87 m	69.6
5′	4.00 ddd, 8.2, 4.0, 2.5 Hz	84.0
6′	3.74 d, 12.1 Hz;<br>3.51 dd, 12.1, 4.0 Hz	60.7

Supplementary Table 2. NMR data of Angustmycin A in DMSO, 400MHz.

NO.	^{1}H (δ, multi)	^{13}C (δ)
2	7.78 s	139.1
4		148.6
5		156.6
6		120.4
8	8.14 s	152.6
1′	4.18 dd, 12.2, 6.0 Hz;<br>3.97 dd, 12.2, 5.1 Hz	61.4
2′		98.9
3′	5.06 t, 4.2 Hz	72.0
4′	4.30 brs	70.2
5′		162.7
6′	4.53 brs; 4.19 t, 2.2 Hz	84.0

Supplementary Table 3. Deduced functions of the open reading frames in the *agm* gene cluster.

Protein	aa #	Protein function	Homolog, origin	Identity, similarity (%)	Accession no.
AgmT1	435	MFS transporter	BB341_26855, *Streptomyces clavuligerus* F613-1	71, 78	ANW21574
AgmT2	399	MFS transporter	FHS42_001181, *Streptomyces zagrosensis*	55, 72	MBB5934155
AgmR	290	LacI family transcriptional regulator	A6A06_38595, *Streptomyces* sp. CB02923	50, 65	OKI04013
AgmA	193	AMP phosphoribohydrolase	LOG, Oryza sativa *Japonica* Group	54, 68	AK071695
AgmB	224	HAD-Like Hydrolase (Phosphatase)	C5D20_10175, *Rathayibacter toxicus*	51, 66	PPH81338
AgmC	359	D-allulose 6-phosphate pyrophosphokinase	CSH63_04110, *Micromonospora tulbaghiae*	49, 61	AYF26663
AgmD	239	D-allulose 6-phosphate 3-epimerase	AlsE, *E. coli* BL21 (DE3)	31, 52	QJZ14459
AgmE	178	Adenine phosphoallulosyltransferase	CSH63_04120, *Micromonospora tulbaghiae*	63, 74	AYF26665
AgmF	423	Angustmycin C dehydratase	Rv3248c, *Mycobacterium tuberculosis* H37Rv	34, 50	NP_217765

Supplementary Table 4. Validation of the pCHW501*ΔagmR* by sequencing analysis.

Plasmid	Determined sequence	Theoretical sequence
pCHW501*ΔagmR*	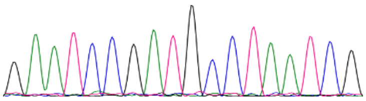 GAA TCC GAT GCC TAA TCG	GAA TCC GAT GCC TAA TCG

Supplementary Table 5. LC-HRMS analysis of the target compounds based on Fig. 5 and Fig. 6.

**Target compound**			**MS ([M+H]$^+$)**	**MS/MS**
**Fig. 5**	AlsE+AgmC	AMP	348.0703	136.0617, 250.0937
	AlsE+Agm(C+A)	Adenine	136.0616	119.0354, 94.0406
	AlsE+Agm(C+A+E)	**5**	378.0806	136.0619, 262.0933, 360.0691
		Adenine	136.0617	119.0353, 94.0408
	AlsE+Agm(C+A+E+B)	**2**	298.1144	136.0617, 280.1026
		Adenine	136.0618	119.0353, 94.0402
	AlsE+Agm(C+A+E+B+F)	**2**	298.1144	136.0616, 280.1028
		**1**	280.1041	136.0617, 262.0934
		Adenine	136.0617	119.0355, 94.0404
	Agm(D+C)	AMP	348.0703	136.0618, 250.0929
	Agm(D+C+A)	Adenine	136.0619	119.0365,94.0331
	Agm(D+C+A+E)	**5**	378.0809	136.0619, 262.0944, 360.0440
		Adenine	136.0618	119.0398, 94.0341
	Agm(D+C+A+E+B)	**2**	298.1148	136.0620, 280.0587
		Adenine	136.0620	119.0341, 94.0342
	Agm(D+C+A+E+B+F)	**2**	298.1143	136.0616, 280.0597
		**1**	280.1039	136.0618, 262.0935
		Adenine	136.0618	119.0352, 94.0409
**Fig. 6**	*alsE+agmCAEBF*	**2**	298.1143	136.0615, 280.1031
		**1**	280.1038	136.0619,262.0927
	agmDCAEBF	**2**	298.1143	136.0617, 280.1021
		**1**	280.1039	136.0618,262.0937
	alsE+agmCAEB	**2**	298.1142	136.0618, 280.1017
	agmDCAEB	**2**	298.1143	136.0619, 280.1029

Supplementary Table 6. Strains and plasmids used in this study.

Strain/Plasmid	Relevant characteristics	Reference or source
**Strain**		
Streptomyces		
S. angustmyceticus JCM 4053	Wild-type of angustmycins producer	JCM
S. decoyicus NRRL 2666	Wild-type of angustmycins producer	NRRL
S. coelicolor M1154	*S. coelicolor* A3(2) derivative	5
M1154::pCHW501	*S. coelicolor* M1154 containing pCHW501	This study
M1154::pCHW501*Δ*agmF	*S. coelicolor* M1154 containing pCHW502	This study
M1154::pSET152	*S. coelicolor* M1154 containing pSET152	This study
Escherichia coli		
E. coli DH10B	F^-, *mcrA*, *Δ*(*mrr-hsdRMS-mcrBC*), φ80d, *lacZΔ*M15, *ΔlacX74*, *deoR*, *recA1*, *endA1*, *araD139*, *Δ*(*ara*, *leu*)7697, *galU*, *galK*, λ^-, *rpsL*, *nupG*	Gibco-BRL
E. coli Rosetta(DE3)/pLysS	F^-, *ompT*, $hsdS_B(r_B\text{-}m_B)$, *gal dcm* λ(DE3) pLysS(Cml^R)	Novagen
E. coli ET12567(pUZ8002)	ET12567 with plasmid pUZ8002(Kan^R)	
E. coli GYJ23	Engineered host for nucleoside production	Unpublished
Mycobacterium smegmatis		
mc^2155	Indicator strain for angustmycins bioassay	ATCC
**Plasmid**		
pEASY-Blunt	pUCori, *lacZ*, f1 ori, *neo*, *bla*	TransGen
pSET152	*aac(3)IV, lacZ, attP(ΦC31), oriT*	6
pET28a	*neo*, rep^{pMB1}, T7 promoter	Novagen
pSJ8	*lac*, MBP, f1 *ori*, *bla*	7
pETDuet	*lacI*, f1 *ori*, T7 promoter, *bla*	Novagen
pCDFDuet	*lacI*, CDF *ori*, T7 promoter, Sm^R	Novagen
pRSFDuet	*lacI*, RSF *ori*, T7 promoter, Kn^R	Novagen
pCHW501	pSET152 derivative carrying a fragment containing whole *agm* gene cluster	This study
pCHW501*ΔagmF*	pSET152 derivative carrying a fragment containing *agm* gene cluster without *agmF*	This study
pCHW501*ΔagmR*	pCHW501 derivative with *agmR* in-frame deleted	This study
pET28a/*agmA*	pET28a derivative carrying a NdeI-EcoRI fragment containing *agmA*	This study
pET28a/*agmB*	pET28a derivative carrying a NdeI-EcoRI fragment containing *agmB*	This study

pSJ8/*agmC*	pET28a derivative carrying a EcoRI-HindIII fragment containing *agmC*	This study
pET28a/*agmD*	pET28a derivative carrying a NdeI-EcoRI fragment containing *agmD*	This study
pET28a/*agmE*	pET28a derivative carrying a NdeI-EcoRI fragment containing *agmE*	This study
pET28a/*agmF*	pET28a derivative carrying a NdeI-EcoRI fragment containing *agmF*	This study
pET28a/$agmF_{\mathrm{H56A}}$	pET28a derivative carrying a NdeI-EcoRI fragment containing $agmF_{\mathrm{H56A}}$	This study
pET28a/$agmF_{\mathrm{D129A}}$	pET28a derivative carrying a NdeI-EcoRI fragment containing $agmF_{\mathrm{D129A}}$	This study
pET28a/$agmF_{\mathrm{K184A}}$	pET28a derivative carrying a NdeI-EcoRI fragment containing $agmF_{\mathrm{K184A}}$	This study
pET28a/$agmF_{\mathrm{D188A}}$	pET28a derivative carrying a NdeI-EcoRI fragment containing $agmF_{\mathrm{D188A}}$	This study
pET28a/$agmF_{\mathrm{H299A}}$	pET28a derivative carrying a NdeI-EcoRI fragment containing $agmF_{\mathrm{H299A}}$	This study
pETDuet/*agmD*+*agmC*	pETDuet derivative carrying a EcoRI-HindIII fragment containing *agmD* and a NdeI-XhoI fragment containing *agmC*	This study
pETDuet/*alsE*+*agmC*	pETDuet derivative carrying a EcoRI-HindIII fragment containing *alsE* and a NdeI-XhoI fragment containing *agmC*	This study
pCDFDuet/*agmA*	pCDFDuet derivative carrying a EcoRI-HindIII fragment containing *agmA*	This study
pCDFDuet/*agmA*+*agmE*	pCDFDuet derivative carrying a EcoRI-HindIII fragment containing *agmA* and a NdeI-XhoI fragment containing *agmE*	This study
pRSFDuet/*agmB*	pRSFDuet derivative carrying a EcoRI-HindIII fragment containing *agmB*	This study
pRSFDuet/*agmB*+*agmF*	pRSFDuet derivative carrying a EcoRI-HindIII fragment containing *agmB* and a NdeI-XhoI fragment containing *agmF*	This study

Supplementary Table 7. Primers used in this study.

Primers	Sequence（5'--3'）
piece1-F	AACAGCTATGACATGATTACGAATTCCCGAACGGGTGCTGGGTT
piece1-R	CGGACGGCGTGCTGTGGT
piece2-F	CCTCTTCGAGGAGGTGGC
piece2-R	AGCTTGGGCTGCAGGTCGACTCTAGATCAGGCGCTGCCCTGGTC
piece3-R	AGCTTGGGCTGCAGGTCGACTCTAGAGGGTGTCATCGGGCAACTC
agm id-F	ACTTCCGGCGCATGGAGA
agm id-R	TCGACGATCAGCGGCTTG
DagmR-First-F	AACAGCTATGACATGATTACGAATTCGCGAACTCCTGGGGCTCT
DagmR-First-R	CATCGGATTCGACGACCT
DagmR-Second-F	GAGGGGCAGGTCGTCGAATCCGATGCCTAATCGCAGACTTCGTG
DagmR-Second-R	TACTGCGGAGCCTCACTCG
agmR-idF	ATGGCGAAGAAAGTTCTG
agmR-idR	TCACCGTCCCGGCGGCCC
agmA-exF	GTCCATATGAAATCGGCCGTCACC
agmA-exR	GGAATTCTCATGCCTCCGCCACCTC
agmB-exF	GTCCATATGACCACCGCACCCCGC
agmB-exR	GGAATTCTCATGAGGTGAACTCCGC
agmC-exF	GGAATTCATGAGTGCCCGGCACCCC
agmC-exR	CCCAAGCTTTCAGTGGGAGCCGGCATG
agmD-exF	GTCCATATGCCGGCTCCCACTGAG
agmD-exR	GGAATTCTCAGGCGGGGGAGAGGGC
agmE-exF	GTCCATATGACGCCCACCCATCCC
agmE-exR	GGAATTCCTAGCCCGGGAAGACGGG
agmF-exF	GTCCATATGACACCCACCATCCAT
agmF-exR	GGAATTCTCAGGCGCTGCCCTGGTC
alsE-exF	GTCCATATGAAAATCTCCCCCTCG
alsE-exR	GGAATTCTTATGCTGTTTTTGCATG
agmA-duet-F	GGAATTCGATGAAATCGGCCGTCACC
agmA-duet-R	CCCAAGCTTTCATGCCTCCGCCACCTC
agmB-duet-F	GGAATTCGATGACCACCGCACCCCGC
agmB-duet-R	CCCAAGCTTTCATGAGGTGAACTCCGC
agmC-duet-F	GTCCATATGAGTGCCCGGCACCCC
agmC-duet-R	CCGCTCGAGTCAGTGGGAGCCGGCATG
agmD-duet-F	GGAATTCGATGCCGGCTCCCACTGAG
agmD-duet-R	CCCAAGCTTTCAGGCGGGGGAGAGGGC
alsE-duet-F	GGAATTCATGAAAATCTCCCCCTCG
alsE-duet-R	CCCAAGCTTTTATGCTGTTTTTGCATG
agmE-duet-F	GTCCATATGACGCCCACCCATCCC
agmE-duet-R	CCGCTCGAGCTAGCCCGGGAAGACGGG
agmF-duet-F	GTCCATATGACACCCACCATCCAT
agmF-duet-R	CCGCTCGAGTCAGGCGCTGCCCTGGTC

Supplementary references

1 Lee, H. C., Kim, J. H., Kim, J. S., Jang, W. & Kim, S. Y. Fermentative production of thymidine by a metabolically engineered *Escherichia coli* strain. *Appl. Environ. Microbiol.* **75**, 2423-2432 (2009).

2 Robert, X. & Gouet, P. Deciphering key features in protein structures with the new ENDscript server. *Nucleic. Acids. Res.* **42**, W320-324 (2014).

3 Zallot, R., Oberg, N. O. & Gerlt, J. A. "Democratized" genomic enzymology web tools for functional assignment. *Curr. Opin. Chem. Biol.* **47**, 77-85 (2018).

4 Zallot, R., Oberg, N. & Gerlt, J. A. The EFI web resource for genomic enzymology tools: Leveraging protein, genome, and metagenome databases to discover novel enzymes and metabolic pathways. *Biochemistry* **58**, 4169-4182 (2019).

5 Gomez-Escribano, J. P. & Bibb, M. J. Heterologous expression of natural product biosynthetic gene clusters in *Streptomyces coelicolor*: from genome mining to manipulation of biosynthetic pathways. *J. Ind. Microbiol. Biotechnol.* **41**, 425-431(2014).

6 Bierman, M. *et al.* Plasmid cloning vectors for the conjugal transfer of DNA from *Escherichia coli* to *Streptomyces* spp. *Gene* **116**, 43-49 (1992).

7 Gao, Y. *et al.* Biosynthesis of 2′-chloropentostatin and 2′-amino-2′-deoxyadenosine highlights a single gene cluster responsible for two independent pathways in *Actinomadura* sp. strain ATCC 39365. *Appl. Environ. Microbiol.* **83**:e00078-17 (2017).
