# Supplementary material for: Efficient biosynthesis of nucleoside cytokinin angustmycin A containing an unusual sugar system
Source: Nat Commun. 2021 Nov 17;12:6633. doi: 10.1038/s41467-021-26928-y (PMC8599513; doi:10.1038/s41467-021-26928-y)
Supplement: Supplementary file 3 — Reporting Summary [file 41467_2021_26928_MOESM3_ESM.pdf]

nature portfolio

Corresponding author(s): Wenqing Chen and Neil P. J. Price

Last updated by author(s): Oct 20, 2021

Reporting Summary

Nature Portfolio wishes to improve the reproducibility of the work that we publish. This form provides structure for consistency and transparency in reporting. For further information on Nature Portfolio policies, see our Editorial Policies and the Editorial Policy Checklist.

Statistics

For all statistical analyses, confirm that the following items are present in the figure legend, table legend, main text, or Methods section.

n/a	Confirmed	
☐	☒	The exact sample size (n) for each experimental group/condition, given as a discrete number and unit of measurement
☐	☒	A statement on whether measurements were taken from distinct samples or whether the same sample was measured repeatedly
☐	☒	The statistical test(s) used AND whether they are one- or two-sided<br>*Only common tests should be described solely by name; describe more complex techniques in the Methods section.*
☒	☐	A description of all covariates tested
☒	☐	A description of any assumptions or corrections, such as tests of normality and adjustment for multiple comparisons
☐	☒	A full description of the statistical parameters including central tendency (e.g. means) or other basic estimates (e.g. regression coefficient) AND variation (e.g. standard deviation) or associated estimates of uncertainty (e.g. confidence intervals)
☒	☐	For null hypothesis testing, the test statistic (e.g. F, t, r) with confidence intervals, effect sizes, degrees of freedom and P value noted<br>*Give P values as exact values whenever suitable.*
☒	☐	For Bayesian analysis, information on the choice of priors and Markov chain Monte Carlo settings
☒	☐	For hierarchical and complex designs, identification of the appropriate level for tests and full reporting of outcomes
☒	☐	Estimates of effect sizes (e.g. Cohen's d, Pearson's r), indicating how they were calculated

Our web collection on statistics for biologists contains articles on many of the points above.

Software and code

Policy information about availability of computer code

Data collection	ESPript (v3.0)
Data analysis	Software used are listed as follows:<br>Flye (v2.8.3)<br>Unicycler (Version: 0.4.9)<br>Hmmer (v3.3.1)<br>MEGA (v3.0)<br>Cytoscape (v3.8.2)<br>MestReNova (v9.0.1-13254)<br>ChemBioDraw (Ultra v12.0)<br>OriginPro 2020 (vSR1 9.7.0.188)<br>Discovery Studio (v3.0)

For manuscripts utilizing custom algorithms or software that are central to the research but not yet described in published literature, software must be made available to editors and reviewers. We strongly encourage code deposition in a community repository (e.g. GitHub). See the Nature Portfolio guidelines for submitting code & software for further information.

Data

Policy information about availability of data

All manuscripts must include a data availability statement. This statement should provide the following information, where applicable:

- Accession codes, unique identifiers, or web links for publicly available datasets
- A description of any restrictions on data availability
- For clinical datasets or third party data, please ensure that the statement adheres to our policy

The two nucleotide sequences reported in this study have been deposited in GenBank under accession no. CP082945 (https://www.ncbi.nlm.nih.gov/nuccore/CP082945) and CP082301 (https://www.ncbi.nlm.nih.gov/nuccore/CP082301), and the two gene cluster sequences have been deposited in GenBank under accession no. MZ151497 (https://www.ncbi.nlm.nih.gov/nuccore/MZ151497) and MZ151498 (https://www.ncbi.nlm.nih.gov/nuccore/MZ151498). The homology structural model was constructed according to the X-ray structure from Protein Data Bank under accession codes 3CE6 (https://www.rcsb.org/structure/3CE6). Other data generated and analyzed in this study are available within the article and the Supplementary Information.

Field-specific reporting

Please select the one below that is the best fit for your research. If you are not sure, read the appropriate sections before making your selection.

- [x] Life sciences
- [ ] Behavioural & social sciences
- [ ] Ecological, evolutionary & environmental sciences

For a reference copy of the document with all sections, see nature.com/documents/nr-reporting-summary-flat.pdf

Life sciences study design

All studies must disclose on these points even when the disclosure is negative.

Sample size	In this study, the fermentation data and kinetics analyses were determined with at least 3 replicates of separate samples. The sample size was determined by the better repeatability and controlled accidental error
Data exclusions	No data were excluded from the analyses.
Replication	All of the biochemical assays of enzymes and their variants, and the metabolite detection of Streptomycete strains were replicated for three times. All attempts at replication were successful.
Randomization	The data in this manuscript mainly include genetic/biochemical investigations and pathway reconstitutions, there were no need to allocate samples into experimental groups in essence. Therefore, no random assay is required for our experimental data.
Blinding	Blinding is not applicable in our study because there were no need to allocate samples into experimental groups in essence.

Reporting for specific materials, systems and methods

We require information from authors about some types of materials, experimental systems and methods used in many studies. Here, indicate whether each material, system or method listed is relevant to your study. If you are not sure if a list item applies to your research, read the appropriate section before selecting a response.

Materials & experimental systems

n/a	Involved in the study	
x		Antibodies
x		Eukaryotic cell lines
x		Palaeontology and archaeology
x		Animals and other organisms
x		Human research participants
x		Clinical data
x		Dual use research of concern

Methods

n/a	Involved in the study	
x		ChIP-seq
x		Flow cytometry
x		MRI-based neuroimaging
